# The pathogen *Moniliophthora perniciosa* promotes differential proteomic modulation of cacao genotypes with contrasting resistance to witches´ broom disease

**DOI:** 10.1186/s12870-019-2170-7

**Published:** 2020-01-02

**Authors:** Everton Cruz dos Santos, Carlos Priminho Pirovani, Stephany Cristiane Correa, Fabienne Micheli, Karina Peres Gramacho

**Affiliations:** 1 Department of Biological Science (DCB), Center of Biotechnology and Genetics (CBG), State University of Santa Cruz (UESC), Rodovia Ilhéus-Itabuna km 16, Ilhéus, Bahia 45652-900 Brazil; 2 Stem Cell Laboratory, Bone Marrow Transplantation Center (CEMO), National Cancer Institute (INCA), Rio de Janeiro, RJ Brazil; 3 CIRAD, UMR AGAP, F-34398, Montpellier, France; 4 Molecular Plant Pathology Laboratory, Cocoa Research Center (CEPEC), CEPLAC, Km 22 Rod. Ilhéus-Itabuna, Ilhéus, Bahia 45600-970 Brazil

**Keywords:** Disease resistance, Plant-pathogen interaction, Proteomics, *Theobroma cacao*

## Abstract

**Background:**

Witches’ broom disease (WBD) of cacao (*Theobroma cacao* L.), caused by *Moniliophthora perniciosa*, is the most important limiting factor for the cacao production in Brazil. Hence, the development of cacao genotypes with durable resistance is the key challenge for control the disease. Proteomic methods are often used to study the interactions between hosts and pathogens, therefore helping classical plant breeding projects on the development of resistant genotypes. The present study compared the proteomic alterations between two cacao genotypes standard for WBD resistance and susceptibility, in response to *M. perniciosa* infection at 72 h and 45 days post-inoculation; respectively the very early stages of the biotrophic and necrotrophic stages of the cacao x *M. perniciosa* interaction.

**Results:**

A total of 554 proteins were identified, being 246 in the susceptible Catongo and 308 in the resistant TSH1188 genotypes. The identified proteins were involved mainly in metabolism, energy, defense and oxidative stress. The resistant genotype showed more expressed proteins with more variability associated with stress and defense, while the susceptible genotype exhibited more repressed proteins. Among these proteins, stand out pathogenesis related proteins (PRs), oxidative stress regulation related proteins, and trypsin inhibitors. Interaction networks were predicted, and a complex protein-protein interaction was observed. Some proteins showed a high number of interactions, suggesting that those proteins may function as cross-talkers between these biological functions.

**Conclusions:**

We present the first study reporting the proteomic alterations of resistant and susceptible genotypes in the *T. cacao* x *M. perniciosa* pathosystem. The important altered proteins identified in the present study are related to key biologic functions in resistance, such as oxidative stress, especially in the resistant genotype TSH1188, that showed a strong mechanism of detoxification. Also, the positive regulation of defense and stress proteins were more evident in this genotype. Proteins with significant roles against fungal plant pathogens, such as chitinases, trypsin inhibitors and PR 5 were also identified, and they may be good resistance markers. Finally, important biological functions, such as stress and defense, photosynthesis, oxidative stress and carbohydrate metabolism were differentially impacted with *M. perniciosa* infection in each genotype.

## Background

The cacao tree (*Theobroma cacao L*.), whose seeds are the raw material for chocolate production, is indigenous to the Amazon and Orinoco rainforests of South America, occurring in tropical climate regions such as Colombia, Mexico, Peru, Caribbean islands as well as African countries [1]. The witches’ broom disease (WBD) of cacao tree, caused by *Moniliophthora perniciosa* (Stahel) Aime Phillips-Mora (2005) [2], is one of the most important cacao diseases, which under favorable environment conditions may cause up to 90% losses of cacao annual production [3].

*Moniliopthora perniciosa* is a hemibiotrophic basidiomycota, that begins its infection as biotrophic pathogens but later switch to a necrotrophic lifestyle [4]. The biotrophic mycelium is monokaryotic, without clamp connection and intercellular growth relying on the nutrients present in the apoplastic for its survival. The Infected plant’s cells become hypertrophied and swelling in shoot apex (green brooms) are noted at 15–25 post-infection [5]. The fungus grows in this manner for about 30 days. Following this biotrophic phase, about 40–45 days post infection, a switch to necrotrophic growth occurs. Necrotrophic fungal hyphae are binucleate with clamp connection and intracellular growth, causing apoptosis and necrosis of infected plant’s cells, provoking death of host tissue. As disease progresses, green and “dry brooms” are fully formed at 60 and 90 days post-infection; respectively [5, 6]. On the dead tissue, the intermittence of dry days followed by rainy days induce the basidiomata production [7, 8], in which, the basidiospores, the only infective propagules, are formed and wind dispersed to the plant infection courts; the meristematic tissue causing symptoms in stems, flower cushions, and pods [9].

Studies on the *T. cacao* x *M. perniciosa* pathosystem are mainly related to sequencing and gene expression, such as the *M. perniciosa* genome [10], genome sequencing and effectorome of six isolates of *Moniliophthora* spp. from different hosts [11], *M. perniciosa* cDNA sequencing of different stages in its life cycle [12]. Also, the cDNA library of the *T. cacao* x *M. perniciosa* pathosystem [13], as well as transcriptomic profiling during biotrophic interaction between *T. cacao* x *M. perniciosa* [14]. Regarding to *T. cacao*, a data bank of expressed sequence tags (ESTs) has been developed [15] and the complete genome of two cacao genotypes, Matina (https://www.cacaogenomedb.org/) and Criollo [16], are publicly available. The above studies have revealed that the quantitative differences of gene expression in *T. cacao* in response to *M. perniciosa* may be a consequence of faster activation of host gene defenses that halts pathogen development with distinct temporal and functional patterns in response to fungal life stages. Incompatible interactions shows strong expression of defense-related genes in the very early stages of infection, 48 and/or 72 h post infection, when shoot apex exhibits no macroscopic symptoms. As well as in the early (45 days post infection) necrotrophic stage of the cacao x *M. perniciosa* interaction.

Despite their importance, in a post-genomic context, these studies alone are not enough to the complete understanding of the *M. perniciosa* and *T. cacao* interaction [17]. Proteomic approaches have the advantage to study the final product of gene expression (proteins), helping to comprehend what is really being translated, as well as its accumulation profile.

The accumulation of proteins can be influenced by post transcriptional and translational alterations, which is associated with the low correspondence to the expression levels of its coding genes [18]. Proteomic studies are being widely applied evidencing alterations in the plant proteome during infection, therefore allowing identification of important proteins expressed in the host in response to the pathogen’s attack [19–21]. Proteomic studies were successfully conducted in other pathosystems, such as the tomato x *Fusarium oxysporum* where several proteins linked to disease resistance were identified in the xylem [22], as well as the proteomic profile of *Arabidopsis thaliana* x *Alternaria brassicicola*, that showed *A. thaliana* cell cultures defense response caused by pathogen-derived elicitors added in the growth medium [23].

The two-dimensional electrophoresis (2D-PAGE) followed by mass spectrometry was already used in studies involving *M. perniciosa*, such as the proteomic analyses of in vitro basidiospores germination [24], protein networks of basidiospores [25] and evaluation of *M. perniciosa* isolates differing in virulence on cacao seedlings [26]. Similarly, cacao proteomic studies such as protocol optimization to protein extraction [27], somatic and zygotic embryogenesis evaluation [28], seeds development and fruit ripening [29] and phylloplane protein identification in different genotypes of cacao [30] were also carried out. However, our understanding of the *T. cacao* x *M. perniciosa* interaction at the proteomic level is still very limited. Thus, the aim of this study was to increase knowledge of the proteomic alterations of two cacao genotypes contrasting to resistance against WBD in the early stages of disease development, 72 h and 45 days post-inoculation with *M. perniciosa*. We identified more than 500 proteins, involved in important biologic functions such as metabolism, energy, defense and oxidative stress, that showed differences in expression patterns between the two genotypes. The resistant genotype was associated with high diversity of expressed proteins related to stress and defense, oxidative stress, and a strong mechanism of detoxification, that were mostly repressed in the susceptible genotype. We also identified proteins with important roles against fungal plant pathogens, such as chitinases, trypsin inhibitors and PR 5. Such proteins could be useful resistance markers. As far as we know, this is the first study to report the proteomic response of resistant and susceptible cacao genotypes in early stages of the biotrophic and necrotrophic stages of cacao x *M. perniciosa* interaction, using 2D-PAGE and liquid chromatography–mass spectrometry (LC-MS/MS) approaches.

## Results

### Infection of *Theobroma cacao* seedlings with the pathogen *M. perniciosa*

In order to better understand the proteomic alterations in *T. cacao* genotypes contrasting to resistance against WBD during infection, three to 4 weeks old seedlings of both resistant (TSH1188) and susceptible (Catongo) genotypes were inoculated with a suspension of basidiospores of *M. perniciosa* and evaluated regarding symptoms and death, following the infection. Shoot apexes were collected from inoculated and non-inoculated (mock inoculated) experiments from both THS1188 and Catongo at 72 h after inoculation, where the first metabolic response related the establishment of biotrophic mycelium begins to happen, and 45 days after inoculation where the fungus mycelium begins to shift from biotrophic to saprophytic-like phase.

The shoot apexes of *T. cacao* plantlets, of resistant (TSH1188) and susceptible (Catongo) genotypes, at 72 h and 45 days post-infection to *M. perniciosa* were submitted protein extraction and proteomic evaluation through 2D-PAGE and liquid chromatography–mass spectrometry. Using these timelines, we focused our study in the early metabolic responses of the biotrophic and necrotrophic stages of the cacao x *M. perniciosa* interaction.

**Fig. 1:**
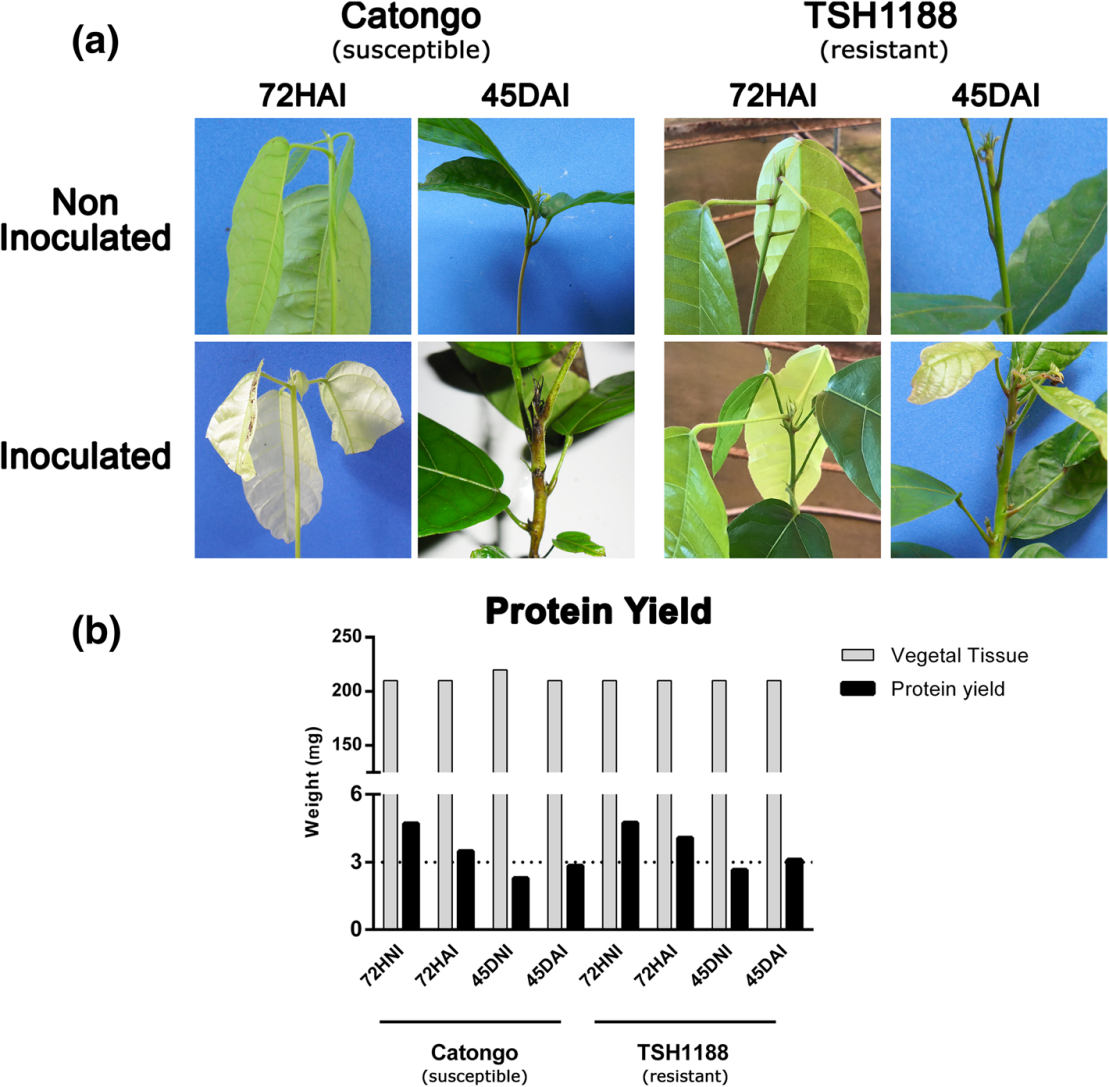
*T. cacao* seedlings inoculated and non-inoculated with *M. perniciosa* and protein yield. **a** *Theobroma cacao* seedlings of Catongo (left) and TSH1188 (right), inoculated and non-inoculated with basidiospores of *Moniliophthora. perniciosa* at 72HAI (hours after inoculation) and 45DAI (days after inoculation). Typical symptoms of WBD (stem swellings), characteristic of fungal biotrophic phase was observed in both genotypes at 45DAI. **b** Protein total yield from 0.2 g of plant tissue of Catongo and TSH1188 genotype, inoculated (72HAI and 45DAI) and non-inoculated (72HNI and 45DNI) with basidiospores of *M. perniciosa*

Infection symptoms following the inoculation with *M. perniciosa* were observed weekly. Discoloration and swelling of the shoot apex, as well as internode elongation at 15 days after inoculation (DAI). At 60DAI fully green broom formation was visualized in 82.45% of the susceptible plants whereas in the resistant genotype brooms incidence was 41%, but of small size diameter. At 45DAI leaf tip burning was noticed in both genotypes (Fig. 1a). At the end of the experiment, after 95 days of symptoms observation, the susceptible genotype, Catongo, exhibited around 90% of diseased plants (55.4% dead and 35% symptomatic plants) and 9% of asymptomatic plants, whereas plantlets of the resistant genotype, TSH1188, had 48% of diseased incidence (7% of dead plants and 41% of symptomatic plants) and 52% of asymptomatic plants. Control plants did not show any symptom. Total protein averaged yield was 3538.84 μg (Fig. 1b) and varied from 3824 to 7683 μg. μL-1; the highest yield was observed at 72HAI for both genotypes.

### Protein profiles analysis in response to *M. perniciosa* infection

**Fig. 2:**
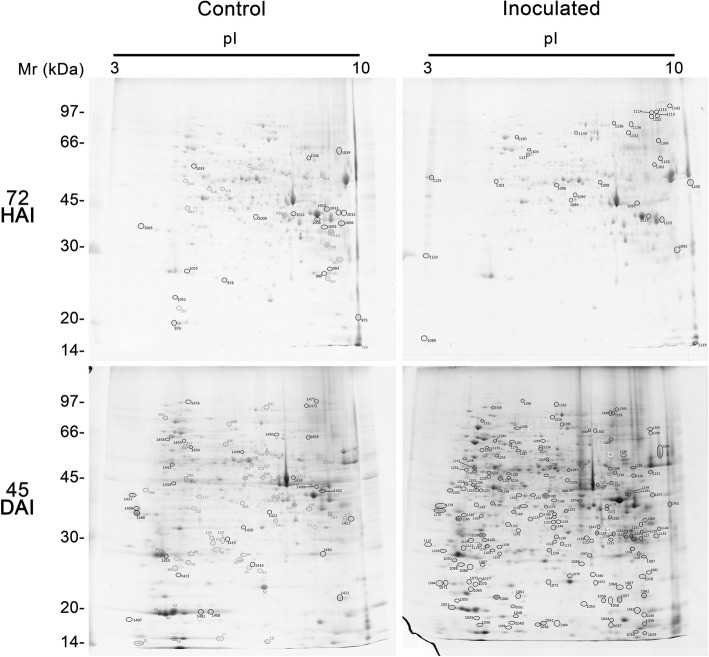
Representative 2D gels of proteins extracted from shoot apexes of TSH1188. Inoculated and non-inoculated (control) cacao genotypes collected at 72HAI and 45DAI post-infection with *M perniciosa*. Total proteins extract (500 μg) were focused on IPG strips (13 cm), pH ranging from 3 to 10 NL, separated by SDS-PAGE (12.5%) and stained with CBB G-250. Circles indicate protein spots identified. Spots number corresponds to protein indicated at Table 1 and Additional file 4

**Fig. 3:**
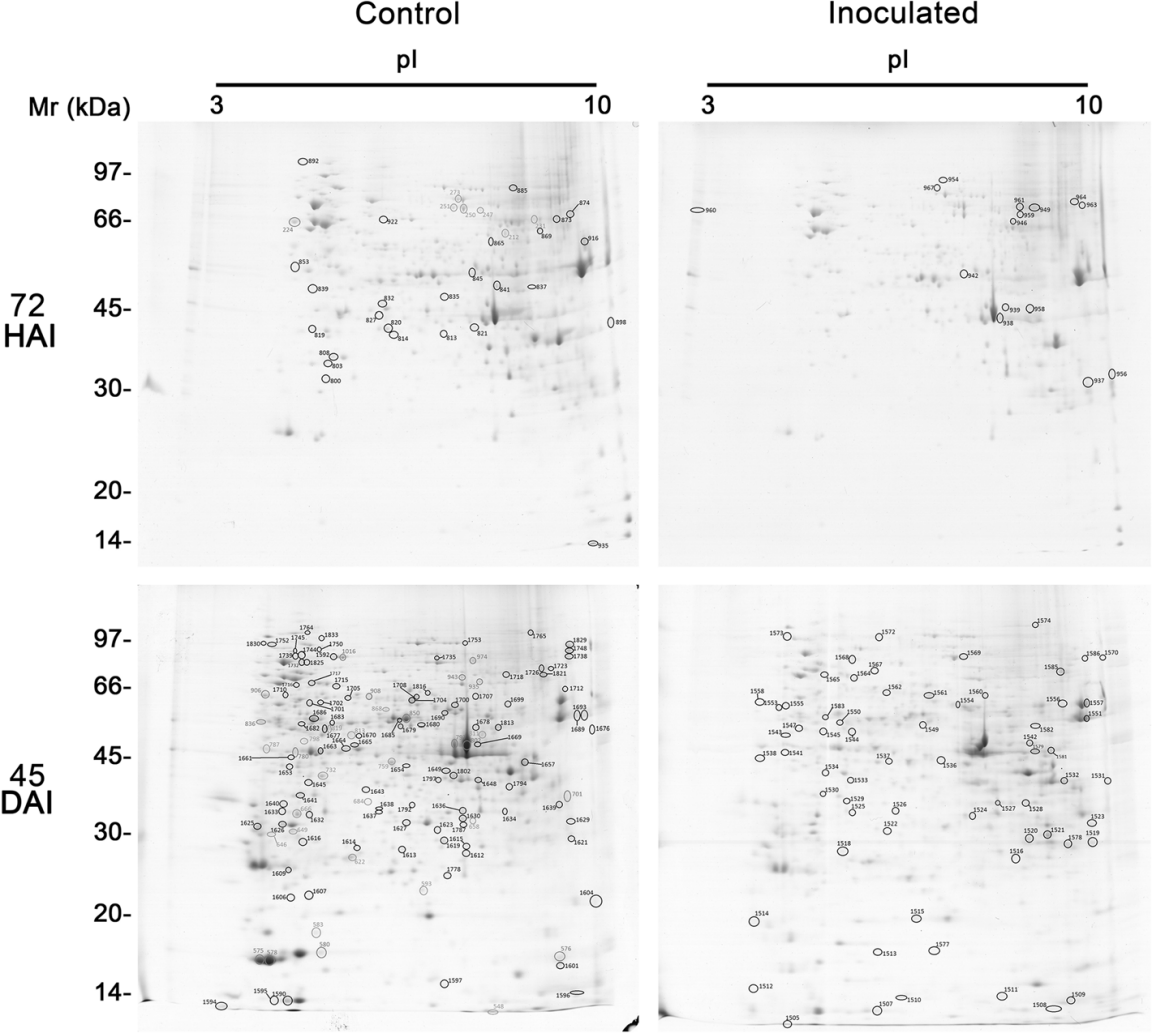
Representative 2D gels of proteins extracted from shoot apexes of Catongo. Inoculated and non-inoculated (control) cacao genotypes collected at 72HAI and 45DAI post-infection with *M perniciosa*. Total proteins extract (500 μg) were focused on IPG strips (13 cm), pH ranging from 3 to 10 NL, separated by SDS-PAGE (12.5%) and stained with CBB G-250. Circles indicates protein spots identified. Spots number corresponds to proteins indicated in the Table 2 and Additional file 5

**Fig. 4:**
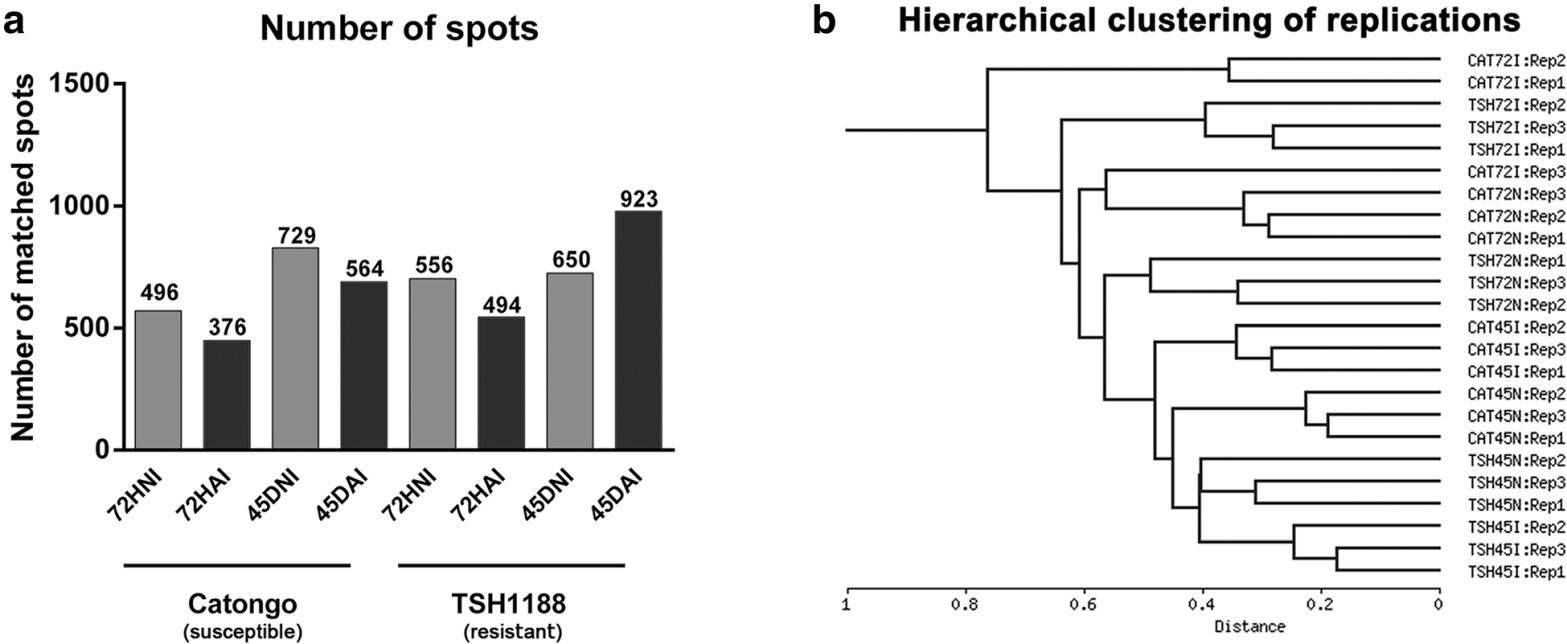
Spot detection and hierarchical clustering of gel replicates. **a** Total number of common spots detected in each treatment performed by Image Master 2D Platinum software 7.0 on 2D gels triplicates images. Spot detection was made by matching the experimental triplicates of each treatment from TSH1188 and Catongo in inoculated conditions (72HAI and 45DAI) and non-inoculated conditions (72HNI and 45DNI). **b** Hierarchical clustering indicating the similarity between experimental replicates based on spot intensity values. This analysis was performed using the NIA array analysis tool software

The two-dimensional gel electrophoresis analysis of the different stages of WBD in two cacao genotypes, TSH1188 (Fig. 2) and Catongo (Fig. 3), with differential phenotypical response to *M. perniciosa* infection, allowed to characterize protein dynamics involved in the disease development. Differential metabolism with specific differential protein expression was observed at each stage, as well as those in common during the developmental process. Infected genotypes were compared with their respective controls. The gel replicates among treatments, which comprised two genotypes (TSH1188 and Catongo) and two collection times (72 HAI and 45 DAI), on inoculated and non-inoculated tissues were equally well resolved, with no significant differences observed in protein yield, reproducibility and resolution (Additional file 1). In both genotypes, more spots were detected in non-inoculated treatments at 72 HAI; this characteristic was more evident in Catongo (Fig. 4a). At 45 DAI, an inversion of that pattern was observed only in the inoculated TSH1188 genotype that, in comparison with the other treatments, showed more detected spots (Fig. 4a). In addition, the hierarchical clustering of replicates regarding to the spots intensity values indicated that a total of 23 of the 24 replicates grouped as expected, showing high similarity of spots between replicates (Fig. 4b). This result seems to endorse the well-resolved reference maps to both control and inoculated treatments of TSH1188 and Catongo genotypes. Differences in fold variation based on the intensity values (*p* ≤ 0.05) of differentially expressed spots were observed through PCA analysis (Additional file 2), that significantly separated the inoculated and non-inoculated treatments, and distinguished the genotype treatments as well. Moreover, these differences and fold variation were significant, showing that the 2DE protein spots were considered regulated in response to infection by *M. perniciosa*. The complete number of spots that were detected in both genotypes and treatments in all analyzed times is showed in Venn diagram (Additional file 3).

### Differentially expressed protein identification

**Fig. 5:**
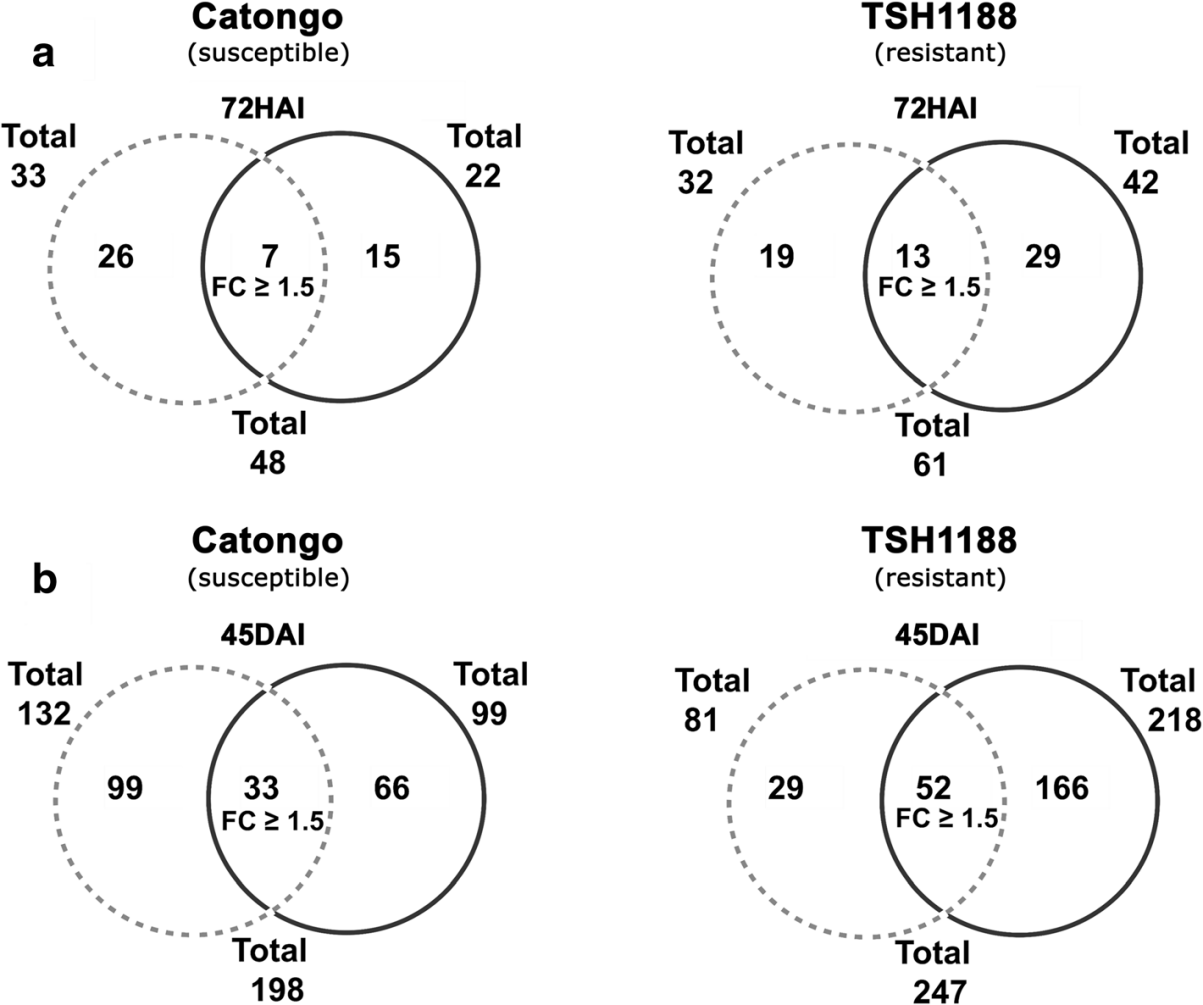
Venn diagrams representing the total number of proteins identified by mass spectrometry in 2D gels from Catongo and TSH1188 cacao genotypes at two time points after inoculation with *M. perniciosa*. **a** 72 h after inoculation (7HAI) and **b** 45 days after inoculation (45DAI). Proteins are discriminated by their occurrence: Gray dashed circles represent non-inoculated treatments, black circles represent inoculated treatments and in the diagrams intersections, the number of significantly common spots altered with Fold change (FC) ≥ 1.5

Before the protein identification, the spots significantly altered (p ≤ 0.05) were selected by matching the images of gels triplicates in silico using Image Master 2D Platinum software. Significantly altered spots were separated as exclusive [spots that appeared only in the inoculated treatment (up regulated proteins) or only in the non-inoculated treatment (down regulated proteins)], and common spots [significantly altered proteins that appeared in both treatments, but with difference in expression levels: fold change (FC) ≥ 1.5]. Through LC-MS/MS approaches, the identities of proteins that were obtained by analyzing the spectra generated with ProteinLynx Global software, were compared against the NCBI data bank and *Theobroma cacao* databank and allowed us to identify a total of 554 protein spots. At 72HAI, 48 and 61 proteins were respectively identified in Catongo and TSH1188, and at 45DAI, 198 and 247 proteins were encountered in Catongo and TSH1188, respectively. More proteins were observed in TSH1188 regardless of the treatment, and most of them were specifically regulated following pathogen infection. However, in Catongo, more proteins were observed in non-inoculated treatments, indicating the overall down regulation of these proteins during pathogen attack in this genotype. Total occurrences of exclusive and common proteins between treatments are illustrated in the Venn diagrams (Fig. 5). List of complete identified proteins and further information can be found at Additional files 4 and 5.

### Functional classification

**Fig. 6:**
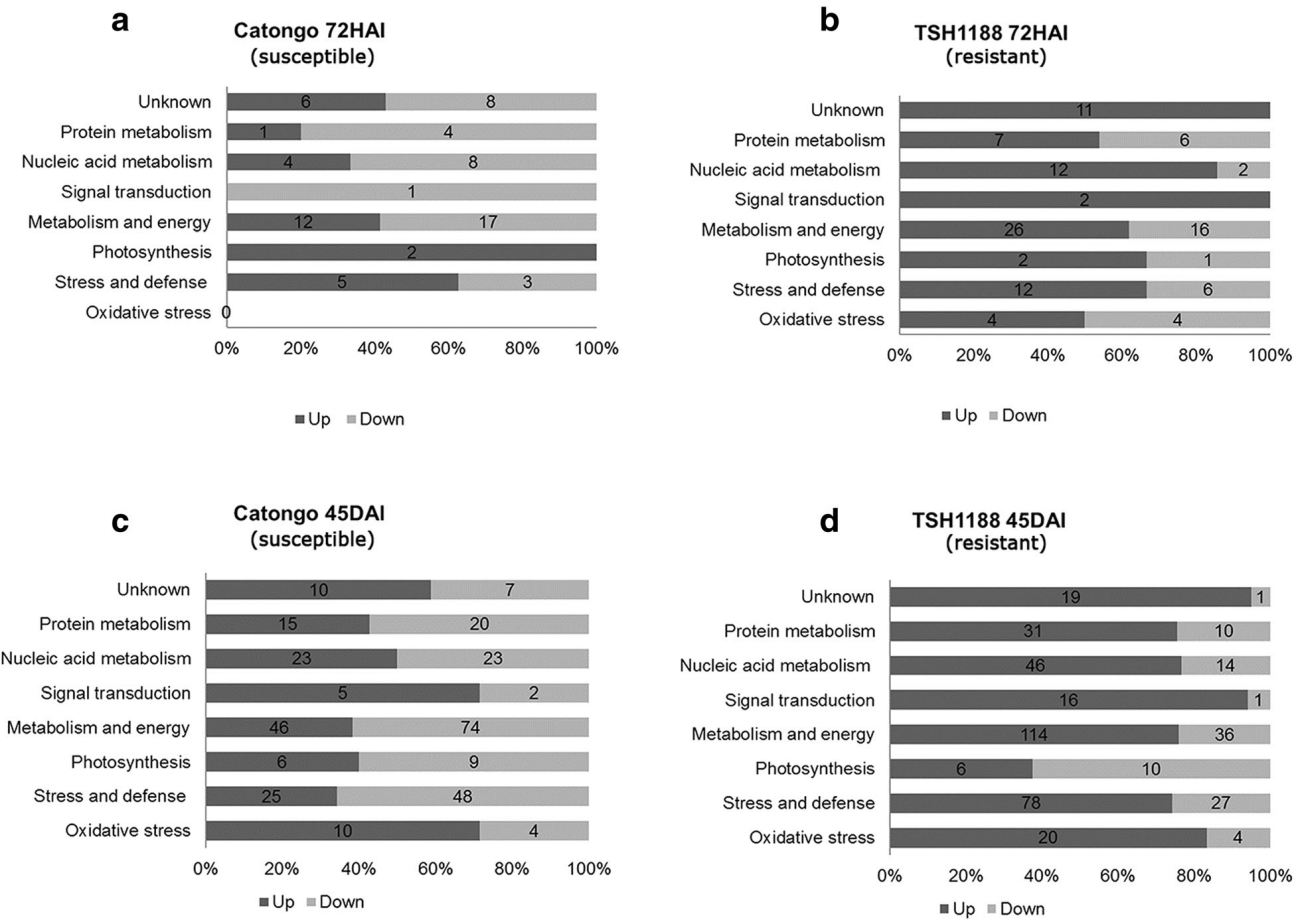
Number of identified proteins discriminated by functional characterization and regulation (up and down). We used the Blast2Go software to divide proteins into eight functional groups: oxidative stress, stress and defense, photosynthesis, metabolism and energy, signal transduction, nucleic acid metabolism, protein metabolism and unknown. Functional characterization of differentially expressed proteins in Catongo (**a**) and TSH1188 (**b**) at 72HAI, and in Catongo (**c**) and TSH1188 (**d**) at 45DAI

Blast2Go tool was used to classify the proteins in 8 functional categories by their biological function. The majority-deregulated proteins in inoculated conditions for both genotypes in both times were associated with energy and metabolism. A significant amount of defense and stress related proteins were observed altered in inoculated treatment of TSH1188 compared to Catongo in 72HAI and 45DAI (Fig. 6). It is interesting to note that TSH1188 showed more up accumulated proteins in response to infection in all functional groups than Catongo. Subcellular localization was also identified for both genotypes (Additional file 6).

### Identified proteins

**Fig. 7:**
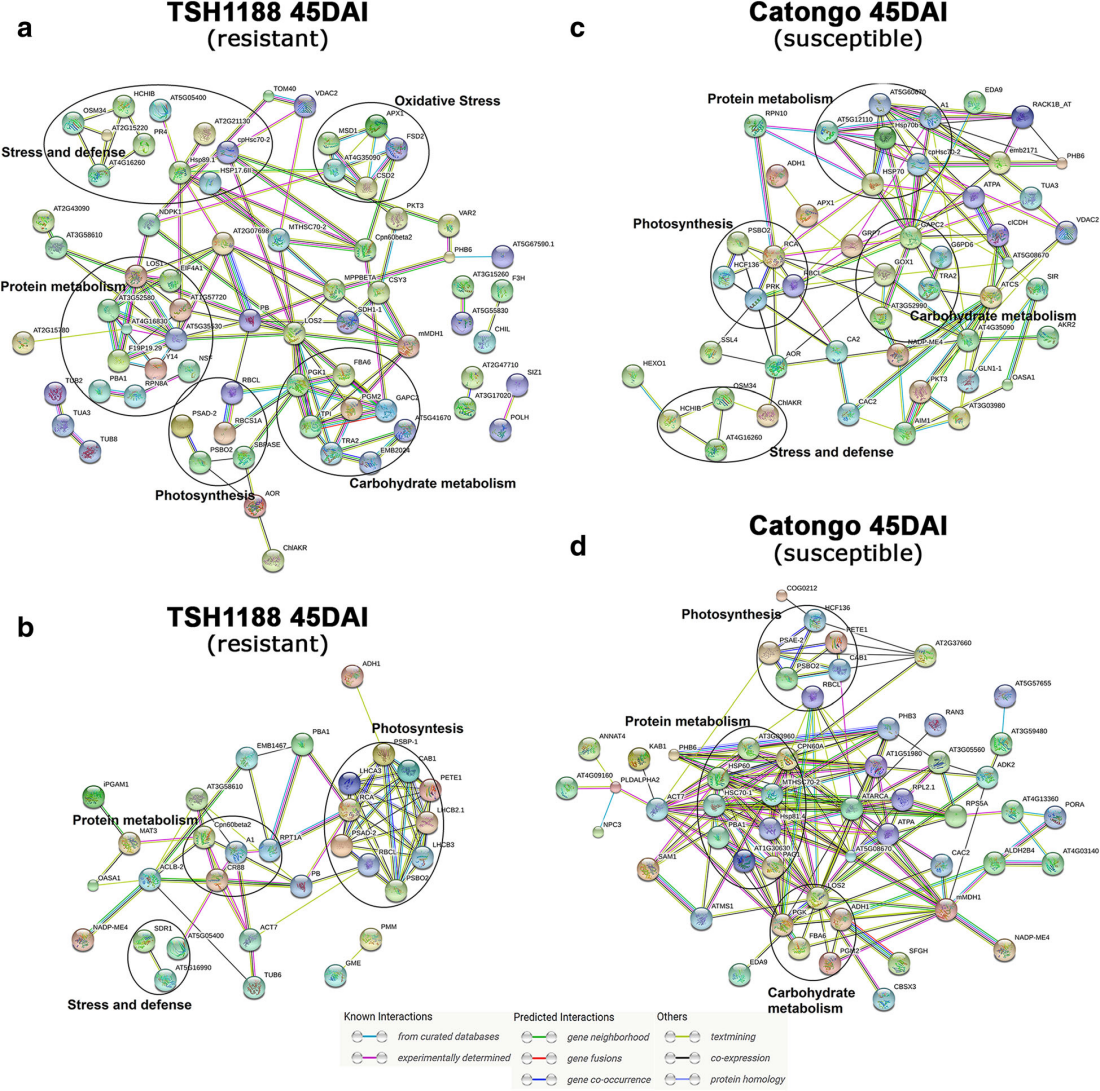
Differentially expressed proteins of TSH1188 and Catongo during interaction with *M. perniciosa* subjected PPI analysis. Networks of up regulated (**a**) and down regulated (**b**) proteins in TSH1188 at 45DAI. Networks of up regulated (**c**) and down regulated (**d**) proteins in Catongo at 45DAI. Dark circles represent highly clustered proteins related to important biological functions. Network nodes represent proteins in which each node represents all the protein by a single, protein-coding gene locus. Small nodes indicate proteins of unknown 3D structure, large nodes indicate proteins which 3D structures are known or predict (can be visualized by close-up the nodes). Different line colors indicate the types of evidence for the associations. Query proteins not connected with network were removed for better visualization

TSH1188 genotype at 72HAI exhibited important oxidative stress proteins up regulated such as glyceraldehyde-3-phosphate dehydrogenase C2 isoform 1 (spot 1123) and isoform 2 (spot 1122), and down regulation of peroxidases (spot 1006, 1005) (Table 1). These groups of proteins were not encountered in Catongo. However, at 45DAI several peroxidases were found up regulated in Catongo (spots 622, 813, 1544, 1531), as well as in TSH1188 (spots 1141, 1132, 1129, 1401, 177: FC + 3.58, 1224, 1222, 1068), including ascorbate peroxidase (spots 96: FC + 1.6 and 1104), which plays an important role in degradation of reactive oxygen species (ROS) and programmed cell death [6] (Table 1 and Table 2). At 72HAI, we also observed that, compared to Catongo, TSH1188 showed more up regulated proteins associated to carbohydrate metabolism such as glyceraldehyde-3-phosphate dehydrogenase (GAPDH) (spot 1123, 1122), glycosyl hydrolase (spot 1106), and putative beta xylosidase alpha L arabinofuranosidase 2 (spot 1120). At 45DAI, proteins in that functional group were markedly up regulated in TSH1188 such as phosphoglycerate kinase 1(spot 1039) which participates in gluconeogenesis and starch biosynthesis (Table 1). Furthermore, although Catongo genotype showed up accumulation of proteins in that functional group at 72HAI, the most altered proteins were down accumulated at 45DAI, such as malate dehydrogenase (spot 1649), enolase (spot 1685), ribokinase (1641) and aldolase (spot 1794, 1648), which indicates metabolism impairment. Photosynthesis proteins were also up regulated in both genotypes at 72HAI, such as ribulose bisphosphate carboxylase/oxygenase activase 1 isoform 1 (spot 1100, 1114) in TSH1188 and a hypothetical protein identified by Basic Local Alignment Search Tool (BLAST) as chloroplast oxygen-evolving enhancer protein 1 (spot 967) in Catongo. Conversely, at 45DAI were observed a greater down regulation of photosynthesis related proteins in both genotypes (Fig. 7, Tables 1 and 2), such as light-harvesting antenna systems (spot 64: FC − 2, spot 73: FC − 1.76, spot 94: FC − 2.29) in TSH1188, and photosystem I and II related proteins (spots 1626, 1595) in Catongo. Defense and stress proteins were more up regulated in TSH1188 at 72HAI, and at 45DAI, the response was much more accentuated. However, Catongo genotype shows overall down regulated pattern at 45DAI (Table 2 and Additional files 3 and 5). In TSH1188 at 72HAI, it was observed, among others, the up regulation of chitinase A (spot 1102), voltage dependent anion channel 2 (spot 381: FC + 1.79)- an important protein related to metabolites exchange, H_2_O_2_ (hydrogen peroxide) accumulation and abscisic acid signaling [31, 32]; down regulation of chaperonin (spot 1033) and one pathogenesis related protein PR-2 a β-1,3-endoglucanases that act against biotic infections (spot 1065). It was noted that at 45 DAI two isoforms of PR-2 were down regulated (spots 1489, 1431), while another two isoforms were up accumulated (spots1170, 1178), also, others were identified up regulated only in TSH1188, such as two PR-4 chitinases (spot 1065, 1097), PR-5 thaumatin (spot 1072), several osmotin type PR-5 (spot 1073, 1060, 1061) and one PR-10.5 (spot 1036). Trypsin inhibitors were down regulated in TSH1188 at 72HAI (spot 974), we also observed the similar pattern at 45 DAI in four isoforms (spot 39: FC − 2, spot 40: FC − 3.5, spot 42: FC -2.8, 1482) although in a low rate compared to 72HAI and as well as to Catongo in both times, which in its turn showed high repression of trypsin inhibitors and others, such as HSP70 (spot 224: FC − 11) at 72HAI. Moreover, three others trypsin inhibitor (spot 1051, 1071 and 1364) showed up regulation in TSH1188 at 45DAI, Catongo instead, presented overall down regulation in proteins associated to stress and defense at this time, although some proteins were up regulated such as voltage dependent anion channel 2 (spot 1578). Others stress response proteins were up regulated in TSH1188 at 45DAI, such as miraculin-like (spot 1056, 1057,1058, 1124), which acts limiting the cellular damage in biotic stress conditions [33], HSP 70 isoforms (spot 224: FC + 7.31284, 1321, 1040), osmotin (spot 1060, 1061,1073), prohibitin (spot 1146), and hydrolases that are expressed in response to fungal molecules (spot 1042, 1037). It’s interesting to note a down regulation of an ankyrin repeat domain-containing protein 2 (spot 266: FC − 3.3) in TSH1188 and its up regulation in Catongo (spot 1538) at 45DAI.

**Table 1:** Differentially Expressed Proteins identified inTSH1188

Spot ID	Identified Protein/Species	UP/DOWN	Fold change^a^	Biologic function^b^	Cellular localization^c^	Time-course
Oxidative stress						
96	ascorbate peroxidase [*Theobroma cacao*]	UP	1.614	O S	Ch P	45DAI
177	Peroxidase superfamily protein [Theobroma cacao]	UP	3.583	O	A	45DAI
1006	Class III peroxidase [Theobroma cacao]	DOWN	–	O	U	72HAI
1052	2-cysteine peroxiredoxin B [Theobroma cacao]	DOWN	–	O S	A Ch	72HAI
1005	Peroxidase 4	DOWN	–	O	U	72HAI
1033	Chaperonin CPN60 2 mitochondrial	DOWN	–	P E N S O	C M V	72HAI
1068	hypothetical protein CICLE_v10000948mg [Citrus clementina]	UP	–	O	Ch M	45DAI
1104	ascorbate peroxidase [Theobroma cacao]	UP	–	O	P Ch	45DAI
1122	Glyceraldehyde-3-phosphate dehydrogenase C2 isoform 2 [Theobroma cacao]	UP	–	E O S	C A Ch M N P	72HAI
1123	Glyceraldehyde-3-phosphate dehydrogenase C2 isoform 1 [Theobroma cacao]	UP	–	E O S	C A Ch M N P	72HAI
1129	Cationic peroxidase 2 precursor [Theobroma cacao]	UP	–	O	U	45DAI
1224	Peroxidase [Theobroma cacao]	UP	–	O	V	45DAI
1401	Class III peroxidase [Theobroma cacao]	UP	–	O	U	45DAI
1421	Peroxidase superfamily protein [Theobroma cacao]	DOWN	–	S O	U	45DAI
1432	Peroxidase superfamily protein isoform 1 [Theobroma cacao]	DOWN	–	O	U	45DAI
1141	Cationic peroxidase 2 precursor [Theobroma cacao]	UP	–	O	U	45DAI
1132	Cationic peroxidase 2 precursor [Theobroma cacao]	UP	–	O	U	45DAI
1129	Cationic peroxidase 2 precursor [Theobroma cacao]	UP	–	O	U	45DAI
1222	Peroxidase [Theobroma cacao]	UP	–	O	V	45DAI
65	Superoxide dismutase [Theobroma cacao]	UP	1.926	O	M	45DAI
17	Copper/zinc superoxide dismutase 2 isoform 1 [Theobroma cacao]	UP	2.129	S O	Ch A	45DAI
1490	Peroxidase superfamily protein isoform 1 [Theobroma cacao]	DOWN	–	O	U	45DAI
Photosynthesis and carbohydrate metabolism						
73	Chlorophyll a-b binding protein 3, chloroplastic [Theobroma cacao]	DOWN	1.761	Ph	Ch	45DAI
1420	Phosphomannomutase [Theobroma cacao]	DOWN	–	E S P T	C	45DAI
1128	6-phosphogluconate dehydrogenase family protein [Theobroma cacao]	UP	–	E N	U	45DAI
1123	Glyceraldehyde-3-phosphate dehydrogenase C2 isoform 1 [Theobroma cacao]	UP	–	E O S	C A Ch M N P	72HAI
1122	Glyceraldehyde-3-phosphate dehydrogenase C2 isoform 2 [Theobroma cacao]	UP	–	E O S	C A Ch M N P	72HAI
398	Insulinase (Peptidase family M16) protein isoform 1 [Theobroma cacao]	UP	1.56	P E S	V M C N P	45DAI
1411	Photosystem I subunit D-2 [Theobroma cacao]	DOWN	–	Ph	Ch	45DAI
1138	Glycosyl hydrolase superfamily protein [Theobroma cacao]	UP	–	E S	V	45DAI
1100	Ribulose bisphosphate carboxylase/oxygenase activase 1 isoform 1 [Theobroma cacao]	UP	–	S T E Ph O	Ch A	72HAI
206	Aldolase superfamily protein isoform 1 [Theobroma cacao]	UP	1.802	S E	C N M Ch P A	45DAI
353	Amidase family protein isoform 1 [Theobroma cacao]	DOWN	3.979	E	U	45DAI
64	Light-harvesting chlorophyll B-binding protein 3 [Theobroma cacao]	DOWN	2.003	Ph E	Ch	45DAI
1009	Lactate/malate dehydrogenase family protein [Theobroma cacao]	DOWN	–	E S N	A Ch M	72HAI
1039	Phosphoglycerate kinase 1 [Theobroma cacao]	UP	–	E S O N	M C A Ch	45DAI
1038	Sedoheptulose-bisphosphatase [Theobroma cacao]	UP	–	S E T O	Ch	45DAI
1302	Glycosyl hydrolase family 38 protein isoform 1 [Theobroma cacao]	UP	–	E	V A	45DAI
94	Chlorophyll a-b binding protein, chloroplastic [Theobroma cacao]	DOWN	2.291	Ph	Ch	45DAI
1106	Glycosyl hydrolase family protein isoform 1 [Theobroma cacao]	UP	–	E	U	72HAI
1488	hypothetical protein CICLE_v10012049mg [Citrus clementina]	DOWN	–	E S	M A N C P Ch	45DAI
1138	Putative uncharacterized protein	UP	–	S E	Ch P	72HAI
1120	Putative Beta xylosidase alpha L arabinofuranosidase 2	UP	–	E	U	72HAI
Stress and defense						
1057	putative miraculin-like protein 2 [Citrus hybrid cultivar]	UP	–	S	U	45DAI
381	Voltage dependent anion channel 2 [Theobroma cacao]	UP	1.792	E S	M V Ch	72HAI
1127	Voltage dependent anion channel 2 [Theobroma cacao]	UP	–	E S	M Ch P V	45DAI
1321	Heat shock protein 89.1 isoform 1 [Theobroma cacao]	UP	–	P S	Ch M	45DAI
1037	Adenine nucleotide alpha hydrolases-like superfamily protein [Theobroma cacao]	UP	–	S E	P	45DAI
1102	Chitinase A [Theobroma cacao]	UP	–	E S	A	72HAI
1071	21 kDa seed protein, putative [Theobroma cacao]	UP	–	S,E	A P	45DAI
1284	Mitochondrial HSO70 2 isoform 2 [Theobroma cacao]	UP	–	P N S O	M P Ch V	45DAI
1146	Prohibitin 2 [Theobroma cacao]	UP	–	S E	V M Ch	45DAI
16	MLP-like protein 28 [Theobroma cacao]	DOWN	1.69	S	N Ch	45DAI
389	Voltage dependent anion channel 1 [Theobroma cacao]	UP	1.646	E S	M V N Ch P	72HAI
224	Chloroplast heat shock protein 70 isoform 1 [Theobroma cacao]	UP	7.391	P S	M Ch N A	45DAI
1125	Carrot EP3–3 chitinase, putative isoform 1 [Theobroma cacao]	UP	–	E S	A	45DAI
1036	Pathogenesis-related protein 10.5 [Theobroma cacao]	UP	–	S	U	45DAI
1042	Adenine nucleotide alpha hydrolases-like superfamily protein [Theobroma cacao]	UP	–	S E	P	45DAI
1052	2-cysteine peroxiredoxin B [Theobroma cacao]	DOWN	–	O S	A Ch	72HAI
1431	Pathogenesis-related protein P2 isoform 1 [Theobroma cacao]	DOWN	–	S	C	45DAI
1065	Pathogenesis-related protein P2 isoform 1 [Theobroma cacao]	DOWN	–	S	U	72HAI
1170	Pathogenesis-related protein P2 isoform 2, partial [Theobroma cacao]	UP	–	S	C	45DAI
1065	Pathogenesis-related protein PR-4B [Theobroma cacao]	UP	–	S	U	45DAI
52	Abscisic stress ripening protein [Theobroma cacao]	DOWN	8.911	S	U	45DAI
974	21 kDa seed protein [Theobroma cacao]	DOWN	–	S,E	A P	72HAI
39	21 kDa seed protein [Theobroma cacao]	DOWN	2.013	S,E	A P	45DAI
1051	21 kDa seed protein [Theobroma cacao]	UP	–	S,E	A P	45DAI
40	21 kDa seed protein [Theobroma cacao]	DOWN	3.559	S,E	A P	45DAI
1073	Osmotin 34 [Theobroma cacao]	UP	–	S	A	45DAI
1060	Osmotin 34 [Theobroma cacao]	UP	–	S	A	45DAI
1040	17.6 kDa class II heat shock protein [Theobroma cacao]	UP	–	S P	C	45DAI
417	TCP-1/cpn60 chaperonin family protein [Theobroma cacao]	DOWN	1.789	E P S	Ch A N P C	45DAI
1135	class I chitinase [Theobroma cacao]	UP	–	S E T	P V	45DAI
1072	Thaumatin-like protein	UP	–	S	A	45DAI
1033	Chaperonin CPN60 2 mitochondrial	DOWN	–	P E N S O	C M V	72HAI
381	Voltage dependent anion channel 2 [Theobroma cacao]	UP	1.792	E S	M V Ch	45DAI
1065	Pathogenesis-related protein P2 isoform 1 [Theobroma cacao]	Up	–	S	U	45DAI

^a^. No Fold change number indicates exclusive proteins

^b^. Biologic functional characterization performed at Blast2Go software: O = Oxidative stress; S = Stress and defense; Ph = Photosynthesis; E = Metabolism and energy; T = Signal transduction; N = Nucleic acid metabolism; P = Protein metabolism; U = Unknown

^c^. Subcellular localization characterization performed at Blast2Go software: Ch = Chloroplast; M = Mitochondria; C = Cytoplasm; P = Plasma membrane; N = Nucleus; V = Vacuole; A = Apoplast; U = Unknown

**Table 2:** Differentially Expressed Proteins identified in Catongo

Spot ID	Identified Protein/Species	UP/DOWN	Fold change^a^	Biologic function^b^	Cellular localization^c^	Time-course
Oxidative stress						
622	ascorbate peroxidase [*Theobroma cacao*]	UP	1.854	O S	Ch P	45DAI
813	Peroxidase [*Theobroma cacao*]	UP	1.73	O	V	45DAI
1544	Peroxidase [*Theobroma cacao*]	UP	–	O	V	45DAI
1531	Peroxidase 68 [*Theobroma cacao*]	UP	–	O	A	45DAI
1639	Class III peroxidase [*Theobroma cacao*]	DOWN	–	O	A	45DAI
1637	Peroxidase 4	DOWN	–	O	U	45DAI
1657	Peroxidase 4	DOWN	–	O	U	45DAI
Photosynthesis and carbohydrate metabolism						
231	Malate dehydrogenase cytoplasmic	UP	3.354	E S	A V Ch N P C	72HAI
273	Sucrose synthase	UP	2.146	E	U	72HAI
212	Pyrophosphate--fructose 6 phosphate 1 phosphotransferase subunit alpha	UP	1.57	E F N	U	72HAI
946	Rhamnose biosynthesis 1 isoform 1 [*Theobroma cacao*]	UP	–	E N	C	72HAI
967	hypothetical protein CICLE_v10032502mg [*Citrus clementina*]	UP	–	F N E S	Ch A	72HAI
885	Malate dehydrogenase [*Theobroma cacao*]	DOWN	–	E	Ch	72HAI
808	PfkB-like carbohydrate kinase family protein [*Theobroma cacao*]	DOWN	–	E	P	72HAI
916	Beta-glucosidase 44	DOWN	–	E	U	72HAI
1649	Malate dehydrogenase [*Theobroma cacao*]	DOWN	–	S	M Ch A	45DAI
1685	Enolase	DOWN	–	E	C	45DAI
943	NADP-dependent malic enzyme	DOWN	9.172	E N P	C	45DAI
1641	PfkB-like carbohydrate kinase family protein [*Theobroma cacao*]	DOWN	–	E	P	45DAI
1648	Aldolase superfamily protein isoform 1 [*Theobroma cacao*]	DOWN	–	S E	C N Ch P A	45DAI
1678	Phosphoglycerate kinase cytosolic	DOWN	–	E N S	N A P C Ch	45DAI
1569	Aldolase-type TIM barrel family protein isoform 1 [*Theobroma cacao*]	UP	–	E	V N A C	45DAI
787	Aldolase-type TIM barrel family protein isoform 1 [*Theobroma cacao*]	UP	1.612	E	Ch M C	45DAI
868	Glucose-6-phosphate 1 dehydrogenase cytoplasmic isoform	UP	1.593	E	C	45DAI
1626	Photosystem II subunit O-2 [*Theobroma cacao*]	DOWN	–	N S F	A Ch	45DAI
Stress and defense						
250	methionine synthase [*Coffea arabica*]	UP	1.598	E S	A Ch C P	72HAI
2,51	5-methyltetrahydropteroyltriglutamate--homocysteine methyltransferase	UP	2.001	E S	A Ch C P	72HAI
937	Prohibitin 2 [*Theobroma cacao*]	UP	–	E S	M V P CH	72HAI
224	Chloroplast heat shock protein 70 isoform 1 [*Theobroma cacao*]	DOWN	11.11	P S N E	U	72HAI
1551	hypothetical protein CICLE_v10027981mg [*Citrus clementina*]	UP	–	N P S		45DAI
1525	heat shock protein 70B [*Arabidopsis thaliana*]	UP	–	N S P O	C Ch	45DAI
1523	Prohibitin 2 [*Theobroma cacao*]	UP	–	S	M V P Ch	45DAI
583	Osmotin 34 [*Theobroma cacao*]	UP	3.243	S	A	45DAI
1515	Osmotin 34 [*Theobroma cacao*]	UP	–	S	A	45DAI
649	Basic chitinase [*Theobroma cacao*]	UP	2.327	S E T	V P	45DAI
1520	Basic chitinase [*Theobroma cacao*]	UP	–	E S	V P	45DAI
658	Glucan endo 1 3 beta glucosidase basic vacuolar isoform	UP	3.7	S	V	45DAI
1538	Ankyrin repeat domain-containing protein 2 isoform 1 [*Theobroma cacao*]	UP	–	P S	N C Ch P	45DAI
1507	Uncharacterized protein TCM_004731 [*Theobroma cacao*]	UP	–	S	U	45DAI
575	21 kDa seed protein [*Theobroma cacao*]	DOWN	5.567	SE	A P	45DAI
578	21 kDa seed protein [*Theobroma cacao*]	DOWN	6.331	SE	A P	45DAI
580	21 kDa seed protein [*Theobroma cacao*]	DOWN	2.074			45DAI
1578	Voltage dependent anion channel 2 [Theobroma cacao]	UP	–	E S	M V Ch P	45DAI
1621	Prohibitin 3 isoform 1 [*Theobroma cacao*]	DOWN	–	S E	N Ch P	45DAI
1629	Prohibitin 2 [*Theobroma cacao*]	DOWN	–	S	P	45DAI
1735	5-methyltetrahydropteroyltriglutamate--homocysteine methyltransferase	DOWN	–	ES	A P Ch C	45DAI
1590	MLP-like protein 28 [*Theobroma cacao*]	DOWN	–	S	C Ch N	45DAI
1661	MLP-like protein 28	DOWN	–	S	C Ch N	45DAI
1717	Heat shock 70 kDa protein mitochondrial	DOWN	–	P N S O	M P Ch V	45DAI
1825	Heat shock cognate protein 70–1 [*Theobroma cacao*]	DOWN	–	S P	C	45DAI
1732	Heatshock cognate protein 80	DOWN	–	S P	C	45DAI
1816	Acidic endochitinase [*Theobroma cacao*]	DOWN	–	S E	V	45DAI
1693	putative miraculin-like protein 2 [*Citrus hybrid cultivar*]	DOWN	–	SE	A P	45DAI

^a^. No Fold change number indicates exclusive proteins

^b^. Biologic functional characterization performed at Blast2Go software: O = Oxidative stress; S = Stress and defense; Ph = Photosynthesis; E = Metabolism and energy; T = Signal transduction; N = Nucleic acid metabolism; P = Protein metabolism; U = Unknown

^c^. Subcellular localization characterization performed at Blast2Go software: Ch = Chloroplast; M = Mitochondria; C = Cytoplasm; P = Plasma membrane; N = Nucleus; V = Vacuole; A = Apoplast; U = Unknown

### Protein-protein interaction

To investigate the interactions among the differentially expressed proteins, 386 orthologous proteins previously identified in *A. thaliana* from the 554 total proteins identified here, were used to build up PPI network including direct (physical) as well as indirect (functional) associations [34]. Eight interaction networks were predicted analyzing up and down regulated proteins separately for each genotype in both evaluated periods (Fig. 7 and Additional file 7). A complex protein-protein association was observed, mainly at 45DAI in both genotypes, where most proteins showed direct or indirect interaction, through the number of observed nodes. The following processes were overrepresented: oxidative stress, photosynthesis, protein metabolism, stress and defense and carbohydrate metabolism, corroborating with our previous results. Some proteins identified in the PPIs display high number of interactions, including the connection of distinct biological functions (Fig. 7). Thus, those proteins may be key players in general proteomic alterations in the pathosystem of the present study. Some of these were observed in proteins up regulated in TSH1188 45DAI (40S ribosomal protein S3–3, identifier: AT5G35530; elongation factor EF-2, identifier: LOS1, low expression of osmotically responsive genes 2, LOS2); Down regulated proteins of TSH1188 at 45DAI (photosystem II subunit P-1, identifier: PSBP-1; rubisco activase, identifier: RCA; chaperone protein htpG family protein, identifier: CR88; ATP synthase subunit beta Identifier: PB); Down regulated proteins of TSH1888 at 72HAI (60S ribosomal protein L11–2, identifier: AT5G45775; 40s ribosomal protein SA, identifier: P40); Up regulated proteins of TSH1188 at 72HAI (elongation factor 1-alpha, identifier: A1; voltage dependent anion channel 1, Identifier: VDAC1); Down regulated proteins of Catongo at 45DAI (chaperonin-60alpha; identifier: CPN60A; mitochondrial HSO70 2, identifier: MTHSC70–2; low expression of osmotically responsive genes 2, identifier: LOS2; malate dehydrogenase 1, identifier: mMDH1); Up regulated proteins of Catongo at 45DAI (glyceraldehyde 3-phosphate dehydrogenase, identifier: GAPC2; 60S ribosomal protein L12–3, identifier: AT5G60670; citrate synthase 4, identifier: ATCS; rubisco activase, Identifier: RCA). Proteins nodes generated and their correspondents STRING IDs, as well as further information about Biological process (GO) Molecular function and KEGG Pathways, are provided at Additional file 8.

## Discussion

### Proteome alteration observed in TSH1188 differs from Catongo and may be related to resistance

Plants during biologic stress may allocate energy to defense response against pathogens in detriment of other normal functions [35], which is usually observed at the early 48HAI. Accumulation of H_2_O_2_ during the first 72 h in infected shoot apexes [36] and high peroxidase activity in protein extracts from leaves of cacao seedlings [37] were observed in the present pathosystem. These alterations require a physiological cost to host organism that are reflected in the proteome alterations observed at that time, since it was observed that both genotypes showed less detected spots and protein identification at 72HAI (Additional file 3, Figure A) [38, 39]. A similar pattern was observed in 2D-PAGE gels of the strawberry inoculated with *Colletotrichum fragariae* pathosystem [19].

Considering that TSH1188 showed more spots compared to Catongo at both times and the metabolic shift from an inhibitory metabolism at 72HAI to an inductive metabolism at 45DAI (Additional file 3, Figure A and B), it can be inferred that these responses may be associated with disease resistance in this genotype. Also, it seems to be related with up regulation of metabolic framework compared to the overall repressor pattern observed in Catongo, which showed more repressed proteins in both times. These results differ from da Hora Junior and collaborators (2012) [40]. These authors found in this pathosystem, more differentially expressed genes in Catongo in a transcriptomic study of shoot apexes of cacao challenged with *M. perniciosa*. However, these findings cannot be properly compared to the results of the present study because the authors used different collection times from ours: a pool of samples to characterize early stage (24, 48 and 72 h) and samples from 30 and 60 days. Nevertheless, proteomic and transcriptomic studies often have a weak correlation. This divergence can be explained mainly by post-translational modifications that proteins can undergo and directly influence the structure, location, degradation, metabolism, functions in addition to their stability. These modifications may also influence protein abundance, suggesting that the accumulation of proteins is partially determined by the accumulation and degradation of mRNAs [18, 41, 42]. These finds highlight the differences in proteomic response between genotypes and indicates an overall repressive metabolic pattern in Catongo.

### Oxidative stress proteins production is differently controlled between genotypes during infection: TSH1188 shows a strong mechanism of detoxification

Oxidative oxygen species (ROS) such as superoxide O2^−^, hydrogen peroxide (H_2_O_2_) and hydroxyl radical (OH), are known to be toxic for plants, so they are removed by antioxidative enzymes. Nevertheless, they participate in important signaling pathways, such as development, growth, cell death, and mainly in response to biotic and abiotic stress, acting directly against the pathogens [43]. Moreover, they may function as signaling molecules in subsequent defense response [44]. Furthermore, ROS are toxic for both host and pathogens, therefore, the balance between production and removal of ROS are important during stress response [43]. TSH1188 exhibited up regulation of stress oxidative proteins at 72HAI, among them, isoforms GAPDH. The gene coding this protein was predicted involved in this pathosystem, however, in silico confirmation was not achieved [13]. This protein has other important functions besides its participation in glycolytic pathway [45]. Its cysteine residues can be oxidized [46] and act like ROS signaling transducers as observed during abiotic stress in *A. thaliana* [47]. Hydrogen peroxide formation in cacao tissue infected with *M. perniciosa* increases significantly in the first 72HAI in TSH1188 compared to Catongo, which in turn did not vary [40]. It was verified the inhibition of peroxidase 3 and 4 at 72HAI in TSH1188. That fact may be associated with the need of ROS accumulation, which in cacao tissues, is similar to a hypersensitive response (HR) in early infection stage, therefore improving the resistance response and disease control [40].

At 45DAI, TSH1188 showed up regulation of oxidative stress proteins twice as large as Catongo, particularly in proteins related to ROS detoxification (Fig. 6, Table 1 and Additional file 4). This change in pattern, may be associated with the fungus’ shift from biotrophic to saprophytic-like stage which has already started at 45DAI, since clamp connections (characteristic of saprophytic mycelium) have been observed in hyphae of *M. perniciosa* at 45DAI in this pathosystem [5]. Thereby, suggesting that this time point can be considered as a transitional stage. Such mycelium had a remarkable intracellular aggressive growth, leading to tissue death. The stress generated may influence the up regulation burst of oxidative stress proteins observed. Increases in H_2_O_2_ levels at 45DAI were also observed in Catongo [6] and TSH1188 [36], but the increase of H_2_O_2_ in susceptible genotype may be related to promotion of pathogen life cycle [36]. Additionally, our results showed that both genotypes expressed peroxidases. The consistent increase in quantity and diversity in proteins of oxidative stress observed in TSH1188, point out that, in the resistant genotype, this response may be related to a more efficient mechanism of detoxification. This efficiency is required once the burst of ROS in that genotype must be finely controlled to either limit the pathogen infection and minimize the host damage through expression of detoxifying proteins.

### Modulation of carbohydrates metabolism and photosynthesis proteins are required to energy supply during infection in both genotypes

During plant infection, the host may present a reduction on photosynthetic rates to mobilize energy to defense response [48]. This “metabolic cost” has been observed in several pathosystems [19, 49]. The energy required to maintain the responses, results in a greater aid of assimilates, mainly in the form of carbohydrates, however this is a two-edged sword, since the pathogen may use these compounds to self-nutrition, increasing its demand [49]. The up regulation of proteins related to metabolism of carbohydrates observed in our pathosystem may indicate the increase of respiration required. This pattern is a common response and has been observed in the strawberry x *Colletotrichum fragariae* pathosystem [19], maize inoculated with sugarcane mosaic virus [50] and abiotic stress [51].

The levels of soluble sugar increases in the first days of interaction in our pathosystem [52], also, the starch storage levels decrease during early disease stage, being higher in Catongo compared to TSH1188 in the first 15 days, although, at 45DAI, the levels of starch were higher in TSH1188 compared to Catongo [5]. These findings corroborate our results, since we found more up regulated proteins related to metabolism of carbohydrates in TSH1188 at 45DAI, which may be related to more efficient process of hexoses production via starch metabolism to supply the energy requirement at this stage [52]. Notwithstanding, these molecules may be used by the fungus as well, and probably perform important function during the mycelium shift from biotrophic to saprophytic [53].

Both genotypes showed increase in accumulation of proteins related to photosynthesis at 72HAI. Photosynthesis activation can benefit cells through supplying of carbon skeleton and energy to subsequent defense response [54]. The same pattern was observed in the proteomic profile of *Pinus monticola* challenged with *Cronartium ribicola* in compatible and incompatible interaction [55]. Nevertheless, this expression pattern changed at 45DAI when both genotypes showed down regulation of photosynthesis related proteins (Fig. 6). This may be related to the hexoses accumulation that can modulate negatively photosynthesis-associated genes during plant-pathogen interaction [49]. Also, this pattern was already observed in other pathosystem [19]. Moreover, the up accumulation of sugar metabolism proteins observed in our work and the sugar accumulation observed at 45DAI by Sena and colleagues (2014) [5] reinforce that possibility.

### Positive regulation of defense and stress proteins are more robust in TSH1188 genotype during early and late response to infection

Fungal matrix cell wall is composed mainly by chitin, although the host did not produce this molecule, they developed, through evolution, enzymes (e.g chitinases) that are capable to degrade the fungus cell wall during defense response [56]. In the TSH1188 these proteins were detected up regulated at both times and in Catongo, only at 45DAI, evidencing the importance of these proteins during plant pathogen interaction. Transgenic plants expressing chitinases increases its resistance against fungus and other pathogens, once chitin fragments are important pathogen-associated molecular pattern (PAMP), which recognition by hosts results in activation of defense signaling pathways [57]. However, recently Fiorin and colleagues (2018) [58], observed that *M. perniciosa* evolved an enzymatically inactive chitinase (MpChi) that binds with chitin immunogenic fragments, therefore prevents chitin-triggered immunity, evidencing a strategy of immune suppression of the host response by the pathogen. Moreover, PAMPs are expressed during biotrophic development and recent studies showed that Cerato-platanin, a PAMP from *M. perniciosa,* might bind chitin in a high affinity way, leading to an eliciting of plant immune system by fungal chitin released fragments [59, 60]. Furthermore, the ionic channels which trough the PAMPs are recognized [61], are up regulated in TSH1188 at both times and only at 45DAI in Catongo, indicating that in the resistant genotype this mechanism of recognition is activated earlier. This information highlights the complex molecular relation during plant-pathogen interactions.

The resistance response of TSH1188 was also highlighted by the expression of several PRs, mainly at 45DAI, that shows representatives of four families. PRs are a heterogeneous group of proteins with basal expression in plants that are induced mainly during pathogen infection [62, 63]. Gesteira and colleagues (2007) [13] found that PR4 proteins were more represented at the cDNA libraries of TSH1188 in our pathosystem. Moreover, it was also observed, in our present study, the exclusive expression of PR5 in TSH1188, an important protein which has antifungal activity in a large number of fungal species, such as inhibition of spores germination and hyphae growth [64–66], and enhances resistance against plant pathogens, e.g. in transgenic banana x *Fusarium oxysporum* sp. and transgenic potato x *Macrophomina phaseolina* and *Phytophthora infestans* [67, 68]. In addition, data of the present study indicates that Ankyrin repeat domain-containing protein 2 has opposite expression profile between genotypes. This protein is associated with regulation of PRs coding genes and positive regulation of PCD (programmed cell death) [69, 70] which can contribute to the shift of phase of the *M. perniciosa* (biotrophic to saprophytic) by releasing nutrients to fungal mycelium [32]. Furthermore, the trypsin inhibitors, that are natural plant defense proteins against herbivory and related to biotic and abiotic resistance [71, 72], were found isoforms in both genotypes, however, in the cDNA library it was found only in TSH1188 [13]. In addition, only in this genotype were found its up regulation at 45DAI. It is well known that *M. perniciosa* at the biotrophic phase release lytic proteins and proteases that contributes to the pathogenicity [73].

The serine protease inhibitors are widely distributed in living organisms like, fungi, plants, bacteria and humans. Further, it has been related to plant resistance [74]*.* In cacao, the accumulation of these serine protease inhibitors varies in different tissues and genotypes in response to several stress*.* It was highly represented in the RT library of the resistant interaction between *T. cacao* and *M. perniciosa* [13]. These inhibitor shows high abundance in proteomic profile of cacao seed [75], zygotic embryo during development [28] and cacao root submitted to flooding [76]*,* and in cacao leaves also varies in response to heavy metal stress [77]. The most abundant proteinases in the genome of *M. perniciosa* are deuterolysins, a type of fungal metalloproteinases that are similar to bacterial thermolysin [10]. Nevertheless, although this serine protease inhibitor variation is not a specific response to the fungus *M. perniciosa*, we believe that it is an important plant defense response of cacao genotypes to stress, that in this case might act protecting the cacao cells against the fungal hydrolases.

### PPI analysis reveals a global protein network involving important biological functions in response to *M. perniciosa* infection

*M perniciosa* is one of the most important pathogens to cacao trees and to understand the biological processes underlying the proteomic mechanisms during infection is mandatory. Thus, a detailed protein-protein interaction network is highly demanded. Construction of predict PPI networks are challenging for non-model plants, [78, 79] especially when it comes to high-throughput proteomic data. In order to further investigate the resistance and susceptibility of cacao genotypes against *M. perniciosa* we have utilized homology-based prediction to identifying PPI among differentially expressed proteins identified in the pathosystem. It is important to emphasize that, some proteins that were identified as isoforms in the 2D-PAGE electrophoresis, were identified as the same protein in the course of the identification process, which diminish the total number of identifications in the PPI networks due to duplicity of the input.

Proteins are not solitary entities; rather, they function as components of a complex machinery, which functional connections are determinant to general metabolism. The effects of *M. perniciosa* infection on the metabolism of TSH1188 and Catongo are illustrated in the Fig. 7, showing different protein components interacting with their partners in different biological functions, such as stress and defense, oxidative stress, protein metabolism, photosynthesis and carbohydrate metabolism. Surely, these clusters are not separated objects, and they form a global protein network in response to *M. perniciosa* infection, which can help us better understand how these undelaying mechanisms are connected, enabling to predict new functional interactions. This is very important, once available information about PPI in non-model plants is scarce. Similar maps were constructed in other pathosystem, such as, soybean and *Fusarium virguliforme* [80] and may be useful to find out specific proteins that respond to infection [81]. A layer of complexity was added to our study, once we noticed that one or more proteins might be cross-talkers between these biological functions. Such connectivity suggests that there is important PPI related to functional regulation, and they are different between both genotypes during *M. perniciosa* infection. Besides, one of the correlations found between some of these proteins was co-expression. It is known that co-expressed genes are often functionally related, ‘guilt by association’ [82], and may acting in similar pathways. This could result in a set of regulated protein that responds to specific perturbations. Thus, the information generated from PPI analysis, may be helpful to identify new potential disease related proteins and regulation models, aiming the formulation of new hypotheses in order to elucidating the molecular basis of our pathosystem and to improve defense strategies.

These results provide hints about the molecular mechanisms of resistance and susceptibility in the pathosystem. Although these predicted interaction networks still need to be verified and further analyzed in following investigations, it is known that PPI are broadly conserved between orthologous species [83, 84], strengthening the results presented in this paper.

## Conclusions

This is the first study using 2D-PAGE associated with LC MS/MS in investigation of *T. cacao* genotypes differing in response against *M. perniciosa* infection. Here it was possible to follow the proteomic changes resulting from early and late biotrophic phase interaction in both susceptible and resistant models, identifying more than 500 proteins involved in important biological functions. It was also observed that these functions are distinctly altered between genotypes, and possibly is related to resistance in THS1188, which presented a high number and variety of proteins in response to infection compared to Catongo. The study highlighted important proteins that may be related to key functions in resistance such as oxidative stress proteins especially in TSH1188 that showed a strong mechanism of detoxification. Also, positive regulation of defense and stress proteins were more robust in this genotype during early and late response to infection, based on identified proteins with important roles against fungus, such as chitinases, trypsin inhibitors and PR 5. These proteins may be good resistance markers. Finally, biologic important functions such as stress and defense, photosynthesis, oxidative stress and carbohydrate metabolism were differentially impacted in a proteomic level by *M. perniciosa* in each genotype.

**Fig. 8:**
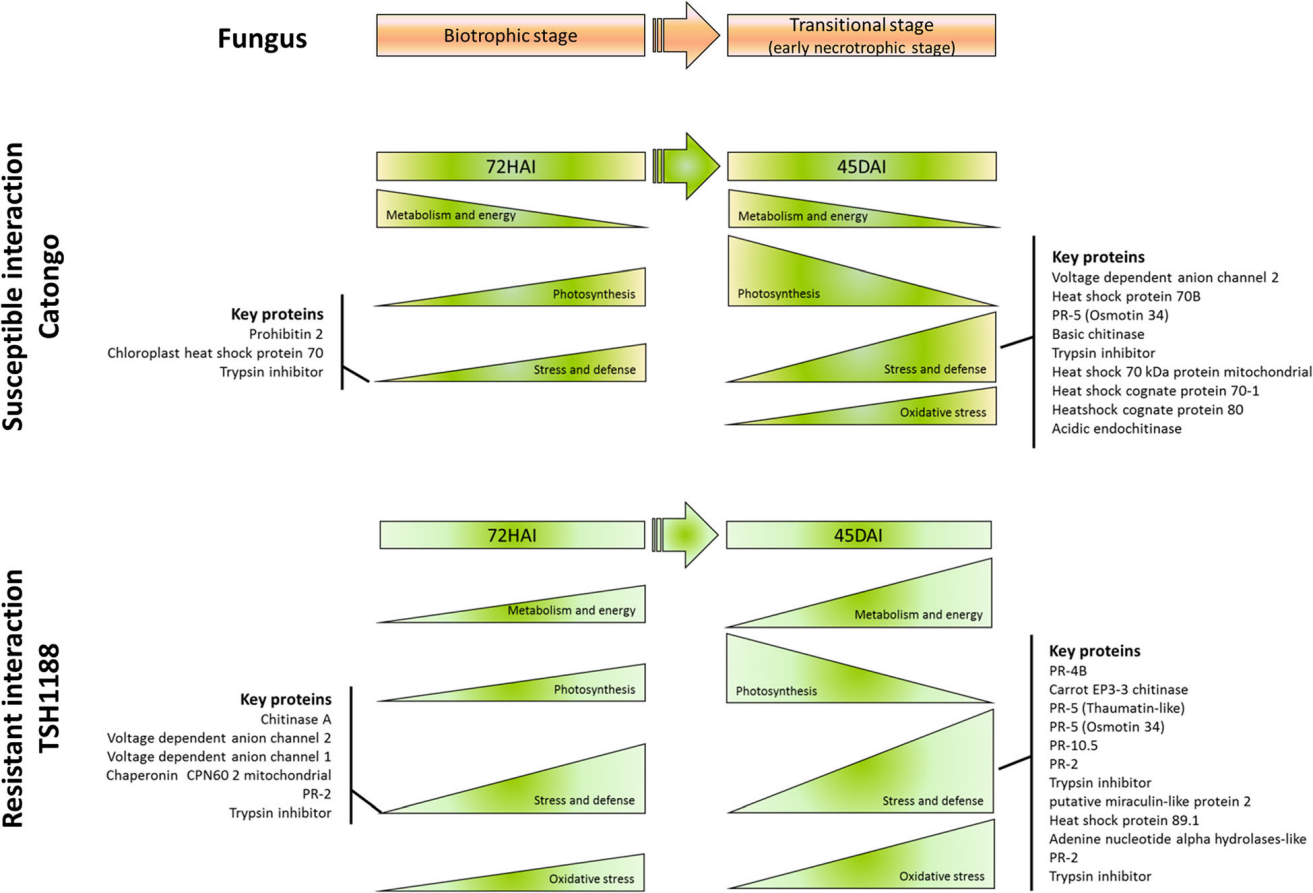
Response model of *T. cacao* genotypes during *M. perniciosa* infection through proteomic approaches. The response of the susceptible (Catongo) and resistant (TSH1188) genotypes to *M. perniciosa* infection vary mainly due the differential protein expression observed by 2D-PAGE-LC/MSMS approach applied in this study. Proteins expression patterns reflect biological functions such as metabolism and energy, oxidative stress, photosynthesis and stress and defense. In general, resistance genotype is mainly related to the early and intense activation of defense pathways/signaling. Nevertheless, the susceptible genotype not only present latter and less intense activation of the mentioned biological functions, but they may be carried out by different proteins from the same biological functions compared to resistant genotype, which can be strongly related to the differential response observed between the evaluated genotypes

Based in these findings, here is suggested a model showing the main alterations observed in both genotypes during infection (Fig. 8). A promising and informative framework of molecular background in both resistance and susceptibility responses of *T. cacao* genotypes during *M perniciosa* infection are provided, highlighting new potential targets for further investigation.

## Methods

### Plant material

The plant material used in this study was chosen based on its demonstrated resistance (TSH1188) and susceptibility (Catongo) to WBD from field progeny trials [85]. Seedlings, derived from open-pollinated pods of all genotypes were obtained from from cacao accessions at the Cacao Germplasm Bank (CGB) of the Cacao Research Center at the headquarters of the Comissão Executiva do Plano da Lavoura Cacaueira (CEPLAC), Ilhéus, Bahia, Brazil (http://www.ceplac.gov.br/). They were planted in a mixture of commercial potting mix (Plantmax®, Eucatex, São Paulo, SP, Brazil) and clay-rich soil, in a 2:1 proportion, and grown in sterile substrate in a greenhouse under natural light and 90% relative humidity until the inoculation day. The International Cacao Germplasm Database – ICGD (http://www.icgd.rdg.ac.uk/) provides further information on TSH 1188 (local name: TSH 1188; accession number: 28′5) and Catongo (local name: SIC 802; accession number: 24).

### Inoculum and inoculation procedures

The shoot apex of the plantlets was inoculated with a basidiospore suspension of inoculum Mp4145*,* from CEPLAC/CEPEC, Ilhéus, Bahia, Brazil, accession number 4145 (CEPLAC/ CEPEC phytopathological *M. perniciosa* collection CEGEN N° 109/2013/SECEXCGEN). The inoculum was prepared as described by Mares and colleagues (2016) [25]. Three to 4 weeks old cacao seedling (plantlets) were subjected to droplet inoculation [5], about 550 seedlings were inoculated in each treatment. Briefly, before inoculation, leaves of seedlings were cut to 2/3 of its length to induce apical growth. Each seedling received a 20 μl suspension of basidiospores in 0.3% water-agar at a concentration of 200.000 spores mL^− 1^. Inoculation was carried out in a moist chamber for 48 h in a dark (23 ± 2 °C temperature; > 97%, relative humidity). After inoculation, the seedlings were transferred to a greenhouse and irrigation for 20 min three times a day until the end of the experiment. The quality of the inoculation was done by assessing by checking the spore germination prior and 24 h after inoculation (≥80% germination). The control seedlings of each genotype were mock inoculated with the same solution without inoculum.

### Experimental design

Each seedling was evaluated weakly for broom type, stem swelling and death. Shoot apexes were collected (around 40) from inoculated and non-inoculated (mock inoculated) experiments from both THS1188 and Catongo at each time point; 72 h after inoculation (72HAI) and 45 days after inoculation (45DAI). All collected shoot apexes were immediately frozen in liquid nitrogen and then lyophilized, followed by protein extraction and proteomic evaluation. The inoculated experiments from each genotype were compared with its matching and non-inoculated control. The remaining plants were used for disease evaluation.

### Protein extraction and dosage

Shoot apexes were submitted to protein extraction using chemical and physical methods to optimize the protein yield in accordance with the protocol developed by Pirovani and colleagues (2008) [27] with modifications. The shoot apexes were macerated and submitted to successive washings of acetone plus trichloroacetic acid solutions followed by sonication steps. A combined process of protein extraction in denaturant conditions using Phenol/SDS buffer was also used. Detailed process can be found in the Additional file 9. Total extracts protein concentration was estimated using the commercial 2D Quant Kit (GE Life Sciences®) following manufacturer’s instructions. Samples concentrations were estimated based on a standard curve with bovine serum albumin (BSA). The protein samples and the curve were prepared in triplicates and read in the Versamax (Molecular Devices) spectrophotometer at 480 nm.

### 1D and 2D gel electrophoresis

The protein profile quality of shoot apexes was evaluated using 20 μg of protein submitted to SDS-PAGE gels (8 × 10 cm, acrylamide 12,5%) in vertical electrophoresis system (Omniphor).

To the 2D analyses, 500 μg of proteins were applied in immobilized pH gradient (IPG) gel strips of 13 cm with pH range of 3–10 NL (Amersham Biosciences, Immobiline™ Dry-Strip). The isoelectric focusing was carried out in the Ettan IPGphor 3 (GE Healthcare) system, controlled by Ettan IPGphor 3 software. Electrofocusing conditions: rehydration time – 12 h at 20 °C; Running - 500Vh for 1 h, 1000Vh for 1:04 h, 8000Vh for 2:30 h and 8000Vh for 40 min. The strips were reduced using equilibrium buffer (urea 6 mol L^− 1^, Tris-HCl pH 8.8 75 mmol L^− 1^, glycerol 30%, SDS 2%, bromophenol blue 0.002%) with DTT 10 mg mL^− 1^ for 15 min, and alkylated using equilibrium buffer with iodoacetamide 25 mg mL^− 1^ for 15 min. Finally, strips were equilibrated with running buffer (Tris 0.25 mol L^− 1^, glycine 1.92 mol L^− 1^, SDS 1%, pH 8.5) for 15 min. The second dimension was carried out in polyacrylamide gels 12.5% (triplicates) and the electrophoresis running were performed in the HOEFER SE 600 Ruby (GE Healthcare) vertical electrophoresis system under the following parameters: 15cmA/gel for 15 min, 40 mA/gel for 30 min and 50 mA/gel for 3 h, or until complete migration of sample trough the gel. After fixation and coloration with colloidal Comassie Brilliant Blue (CBB) G-250, gels were decolorized with distillated water. The digitalization process was made using ImageScanner III (GE Healthcare), the images were analyzed, and the spot detection was made by matching the gels triplicates in silico using Image Master 2D Platinum software (GE Healthcare).

### Statistical analyses

The statistical analysis was made comparing the inoculated to non-inoculated treatments (ANOVA) to identify the differentially (exclusive and common) expressed spots (*p* ≤ 0.05 and ≥ 1.5-Fold change). A multivariate analysis was performed to evaluate the global changes of genotypes in response to infection. Spots intensities values were obtained through digitalization results and were used to find the hierarchical clustering of replicates using NIA *array analysis tool* (http://lgsun.grc.nia.nih.gov/ANOVA/) software. In addition, a principal component analysis (PCA) was performed to identify the phenotypic and genotypic differences between treatments.

### In gel digestion, mass spectrometry and protein identification

The selected protein spots were manually excised from gels and individually bleached, washed, dehydrated and submitted to protein digestion as described by Silva and colleagues (2013) [86] Peptides were resolved by reverse phase chromatography in nanoAcquity UPLC (Ultra Performance Liquid Chromatography) (WATERS), ionized and fragmented in the *Micromass Q-TOFmicro* (WATERS) spectrometer as described by Mares and colleagues (2016) [25]. Spectra were analyzed with *ProteinLynx Global Server* v 2.3 e (*WATERS*) software and compared against the NCBI data bank, using MASCOT MS/MS *Ions Search* (www.matrixscience.com) tool, following the search criteria: Enzyme: Trypsin; Allow up to 1 missed cleavage; Fixed Modifications: Carbamidomethyl (C); Variable Modifications: Oxidation (M); Peptide Tolerance: 30 ppm; MS/MS tolerance: 0.3 Da and 0.1 to fragmented ions. Spectra not identified at NCBI were compared to the *Theobroma cacao* databank (http://cocoagendb.cirad.fr/gbrowse) via *ProteinLynx* using the same criteria. In this work we consider the protein exclusively found in the not inoculated treatments as down regulated, assuming that its accumulation rates were reduced under detection limits as well as, to the protein exclusively found at inoculated treatments considered up regulated.

### Functional annotation

FASTA sequences of identified proteins were obtained in the NCBI databank using the access number generated by MASCOT. The sequences of proteins identified in the *ProteinLyn*x were available in the platform. Biologic function, biologic process and location of proteins were accessed using BLAST2GO (http://www.blast2go.com/) software.

### Protein-protein interaction (PPI)

Before the PPI analyses, orthologous proteins between *T. cacao* and *A. thaliana* of differentially expressed proteins identified in both times to both genotypes during the interaction were searched based on the local alignment of the sequences using BlastP 2.5.0 [87] with shell script comands:-evalue 1E-3 -max_target_seqs 1 -outfmt 6 -num_threads 8. The best hits in *A. thaliana* were considered as orthologous. The PPI analyzes were predicted using *Retrieval of Interacting Genes/Proteins* (STRING) 10.0 version [37] (www.string-db.org). In the software, all analyses were carried against *A. thaliana* database. PPI information was obtained enabling different prediction methods in the software, such as neighborhood, experiments, co-expression, gene fusion, databases, and co-occurrence. Associations were visualized with a medium confidence cutoff (0.400) using *A. thaliana* as standard organism.

## Supplementary information

**Additional file 1. **Example of bidimensional gels (triplicates) highlighting the TSH1188 genotype in 45DAI infected with *M. perniciosa*. Total proteins extract (500 μg) were focused on IPG strips (13 cm), pH ranging from 3 to 10 NL, separated by SDS-PAGE (12.5%) and stained with CBB G-250.

**Additional file 2.** Principal Component Analysis showing the grouping of samples regarding different treatments. In A, biplot for all treatments of the Catongo genotype. B, biplot for all treatments of the TSH1188 genotype. C, biplot for all treatments of the two genotypes analyzed together. Each dot represents a triplicate, named as follows: Initial sequence letters representing the genotypes, followed by the numbers represented by the treatment period, 72HAI and 45DAI and the final letters representing the inoculated (I) and not inoculated (N) treatment.

**Additional file 3.** Venn diagrams representing the total number of spots detected in both genotypes and treatments. Spots are discriminated by their occurrence: Gray dashed circles represent non-inoculated treatments; black circles represent inoculated treatments. In the diagram’s intersections the total number of common spots and the number of common significantly altered with FC ≥ 1.5 are shown.

**Additional file 4.** List of complete differentially Expressed Proteins identified in TSH1188.

**Additional file 5.** List of complete differentially Expressed Proteins identified in Catongo.

**Additional file 6. **Subcellular localization of identified proteins**.** The analysis was performed in the Blast2Go software. Subcellular localization from identified proteins of Catongo (A) and TSH1188 (B) genotypes at 72HAI. Subcellular localization from Catongo (C) and TSH1188 (D) genotypes at 45DAI.

**Additional file 7. **Differentially expressed proteins of TSH1188 and Catongo during interaction with *M. perniciosa* subjected PPI analysis. Networks of up regulated (A) and down regulated (B) proteins in TSH1188 at 72HAI. Networks of up regulated (C) and down regulated (D) proteins in Catongo at 72HAI. Network nodes represent proteins in which each node represents all the protein by a single protein-coding gene locus. Small nodes indicate proteins of unknown 3D structure, large nodes indicate proteins which 3D structures are known or predict (can be visualized by close-up the nodes). Different line colors indicate the types of evidence for the associations. Query proteins not connected with network were removed for better visualization.

**Additional file 8.** Complete list of orthologous proteins subjected to PPI analysis.

**Additional file 9.** Detailed protein extraction method.

## Data Availability

All data generated or analyzed during this study are included in this published article and in its supplementary information files. Seeds were obtained from cacao accessions at the Cacao Germplasm Bank of the Cacao Research Center/Executive Commission of the Cacao Farming Plan —CEPEC/CEPLAC (Ilhéus, Bahia, Brazil; http://www.ceplac.gov.br/). The International Cocoa Germplasm Database – ICGD (http://www.icgd.rdg.ac.uk/) provides further information on TSH 1188 (local name: TSH 1188; accession number: 28′5) and Catongo (local name: SIC 802; accession number: 24). Inoculum was obtained from isolate Mp4145, from CEPLAC/CEPEC, Ilhéus, Bahia, Brazil, accession number 4145 (CEPLAC/ CEPEC phytopathological *M. perniciosa* collection CEGEN N° 109/2013/SECEXCGEN).

## Abbreviations

2D PAGE: Two-dimensional electrophoresis
45DAI: 45 days after inoculation
72HAI: 72 h after inoculation
H_2_O_2_: Hydrogen peroxide
LC-MS/MS: Liquid chromatography–mass spectrometry
PAMP: Pathogen-associated molecular pattern
PCD: Programmed cell death
PPI: Protein-protein interaction
PR: Pathogenesis-related protein
ROS: Oxidative oxygen species
UPLC: Ultra Performance Liquid Chromatography
