# Supplementary figures and images for: The pathogen Moniliophthora perniciosa promotes differential proteomic modulation of cacao genotypes with contrasting resistance to witches´ broom disease
Source: BMC Plant Biol. 2020 Jan 2;20:1. doi: 10.1186/s12870-019-2170-7 (PMC6941324; doi:10.1186/s12870-019-2170-7)

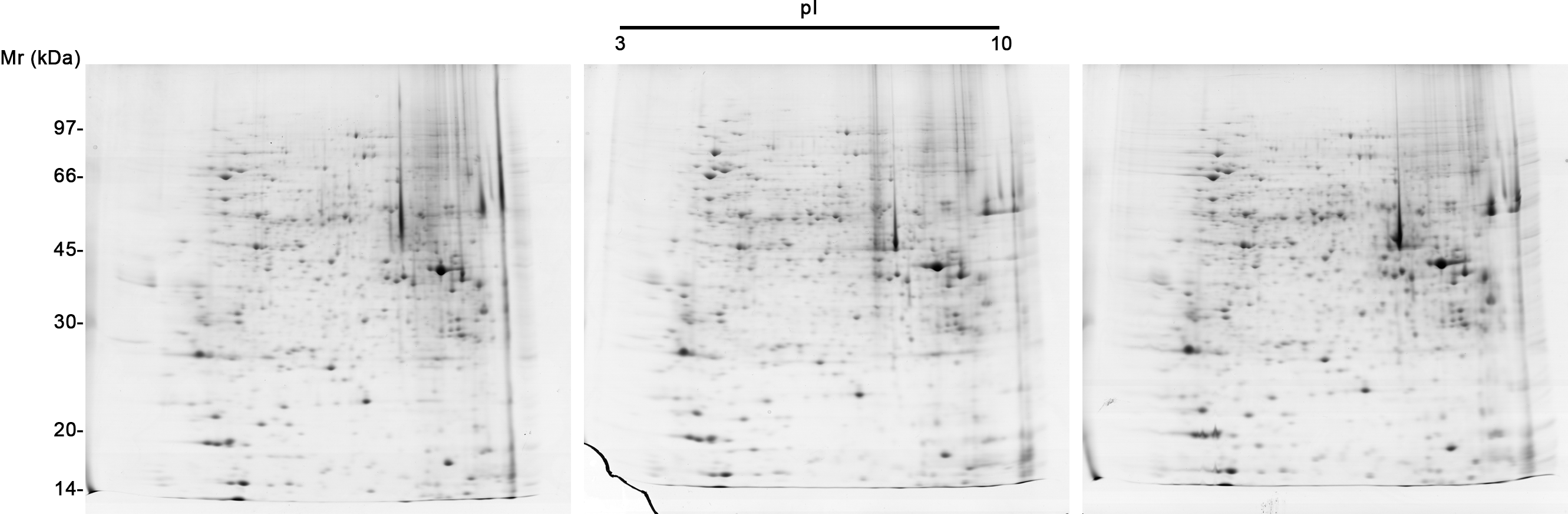

Supplement: Supplementary file 1 — Additional file 1. Example of bidimensional gels (triplicates) highlighting the TSH1188 genotype in 45DAI infected with M. perniciosa. Total proteins extract (500 μg) were focused on IPG strips (13 cm), pH ranging from 3 to 10 NL, separated by SDS-PAGE (12.5%) and stained with CBB G-250. [file 12870_2019_2170_MOESM1_ESM.png]

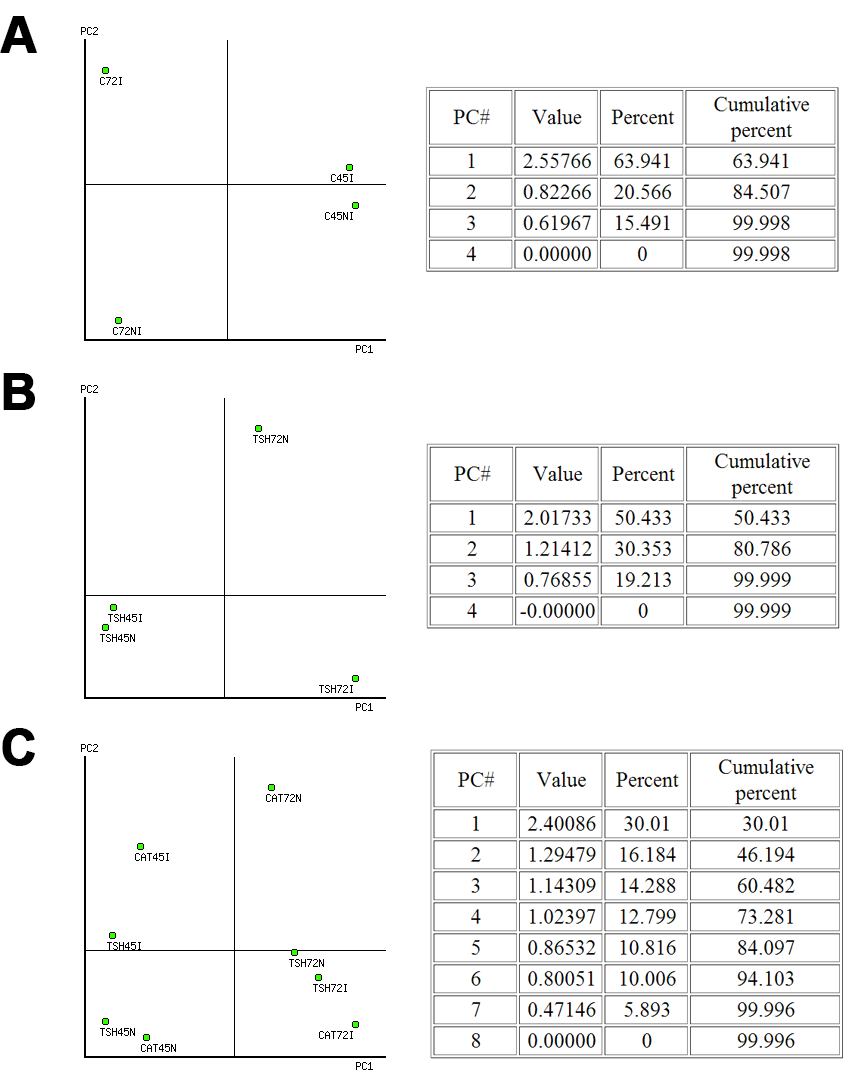

Supplement: Supplementary file 2 — Additional file 2. Principal Component Analysis showing the grouping of samples regarding different treatments. In A, biplot for all treatments of the Catongo genotype. B, biplot for all treatments of the TSH1188 genotype. C, biplot for all treatments of the two genotypes analyzed together. Each dot represents a triplicate, named as follows: Initial sequence letters representing the genotypes, followed by the numbers represented by the treatment period, 72HAI and 45DAI and the final letters representing the inoculated (I) and not inoculated (N) treatment. [file 12870_2019_2170_MOESM2_ESM.tif]

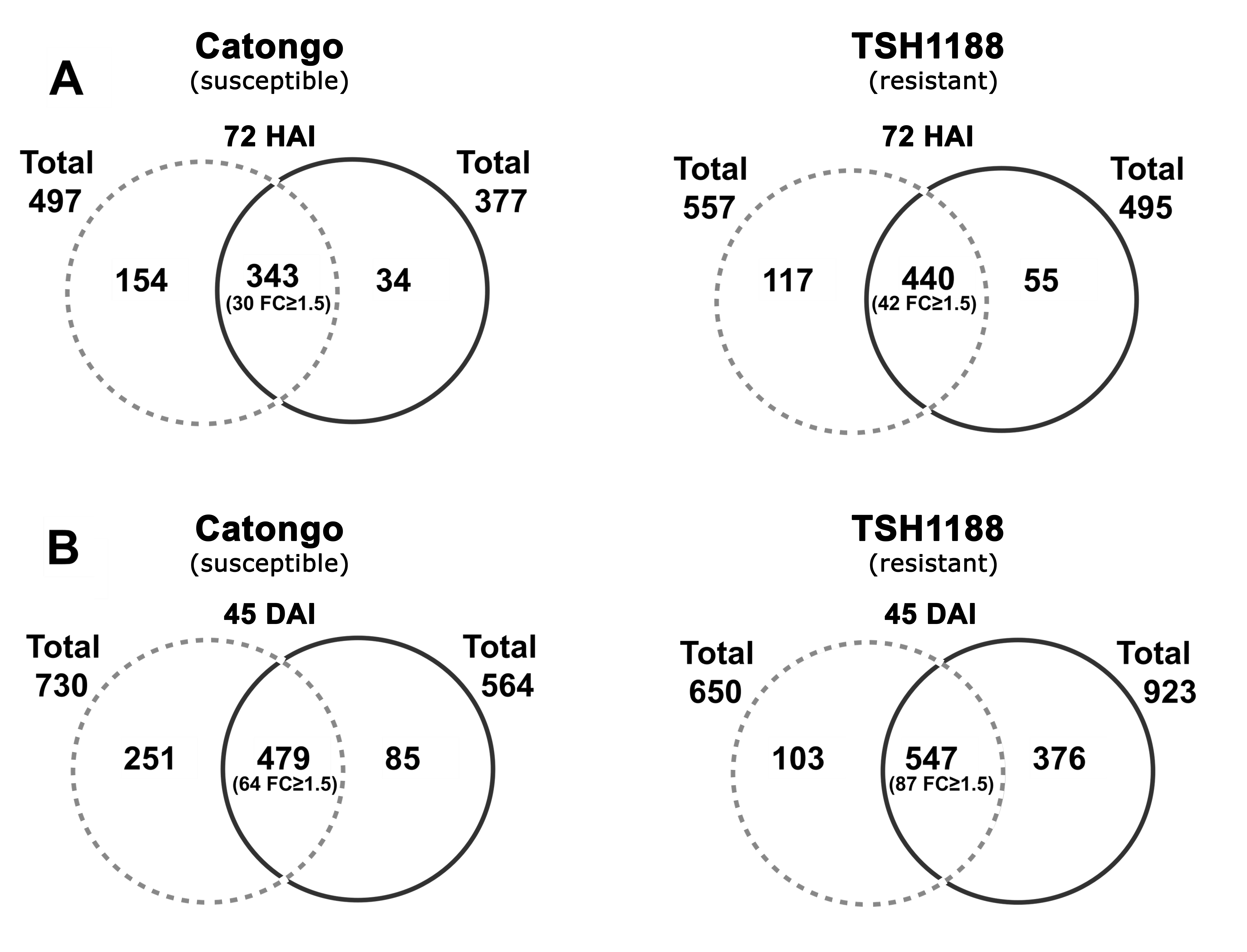

Supplement: Supplementary file 3 — Additional file 3. Venn diagrams representing the total number of spots detected in both genotypes and treatments. Spots are discriminated by their occurrence: Gray dashed circles represent non-inoculated treatments; black circles represent inoculated treatments. In the diagram’s intersections the total number of common spots and the number of common significantly altered with FC ≥ 1.5 are shown. [file 12870_2019_2170_MOESM3_ESM.tif]

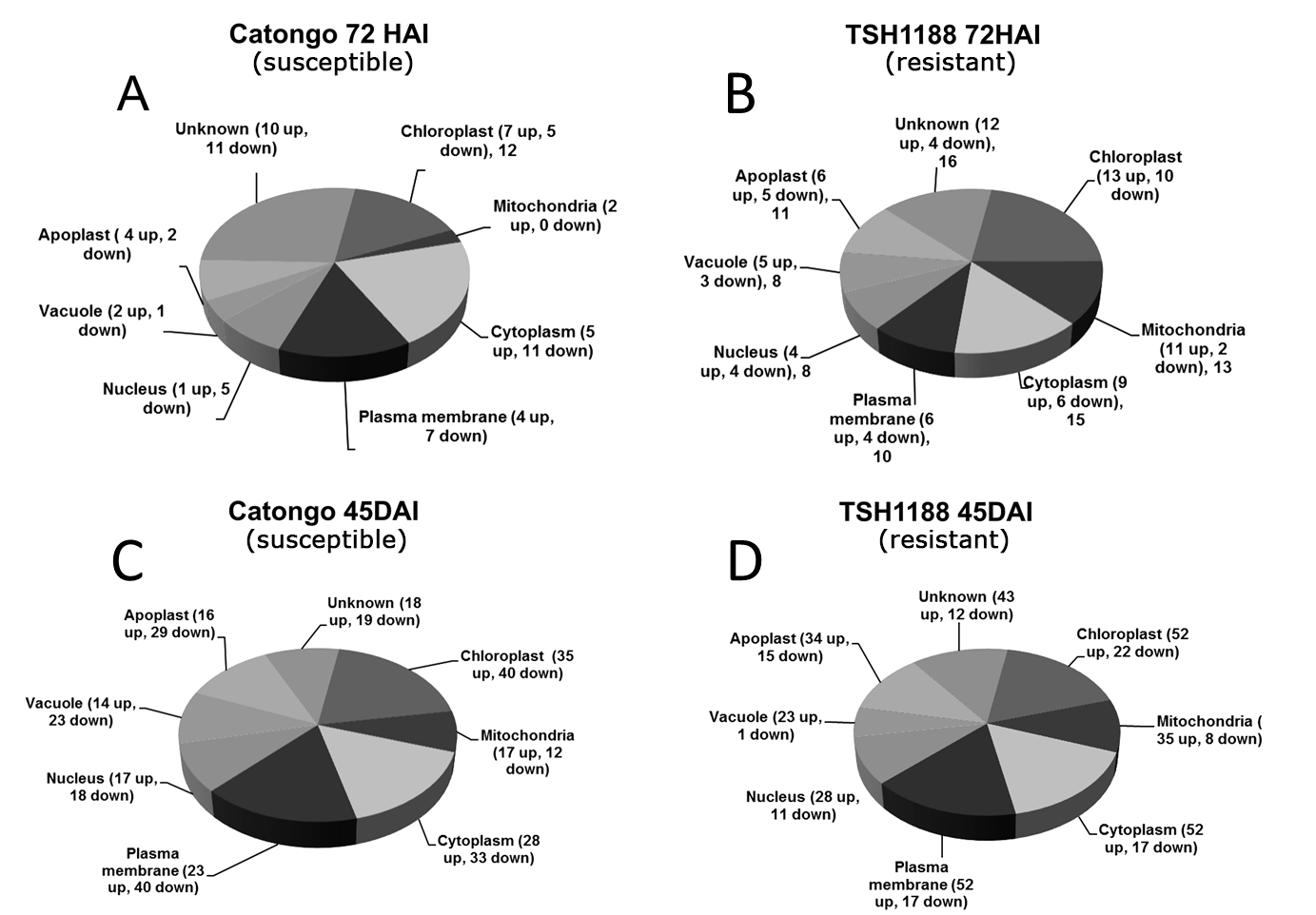

Supplement: Supplementary file 6 — Additional file 6. Subcellular localization of identified proteins. The analysis was performed in the Blast2Go software. Subcellular localization from identified proteins of Catongo (A) and TSH1188 (B) genotypes at 72HAI. Subcellular localization from Catongo (C) and TSH1188 (D) genotypes at 45DAI. [file 12870_2019_2170_MOESM6_ESM.tif]

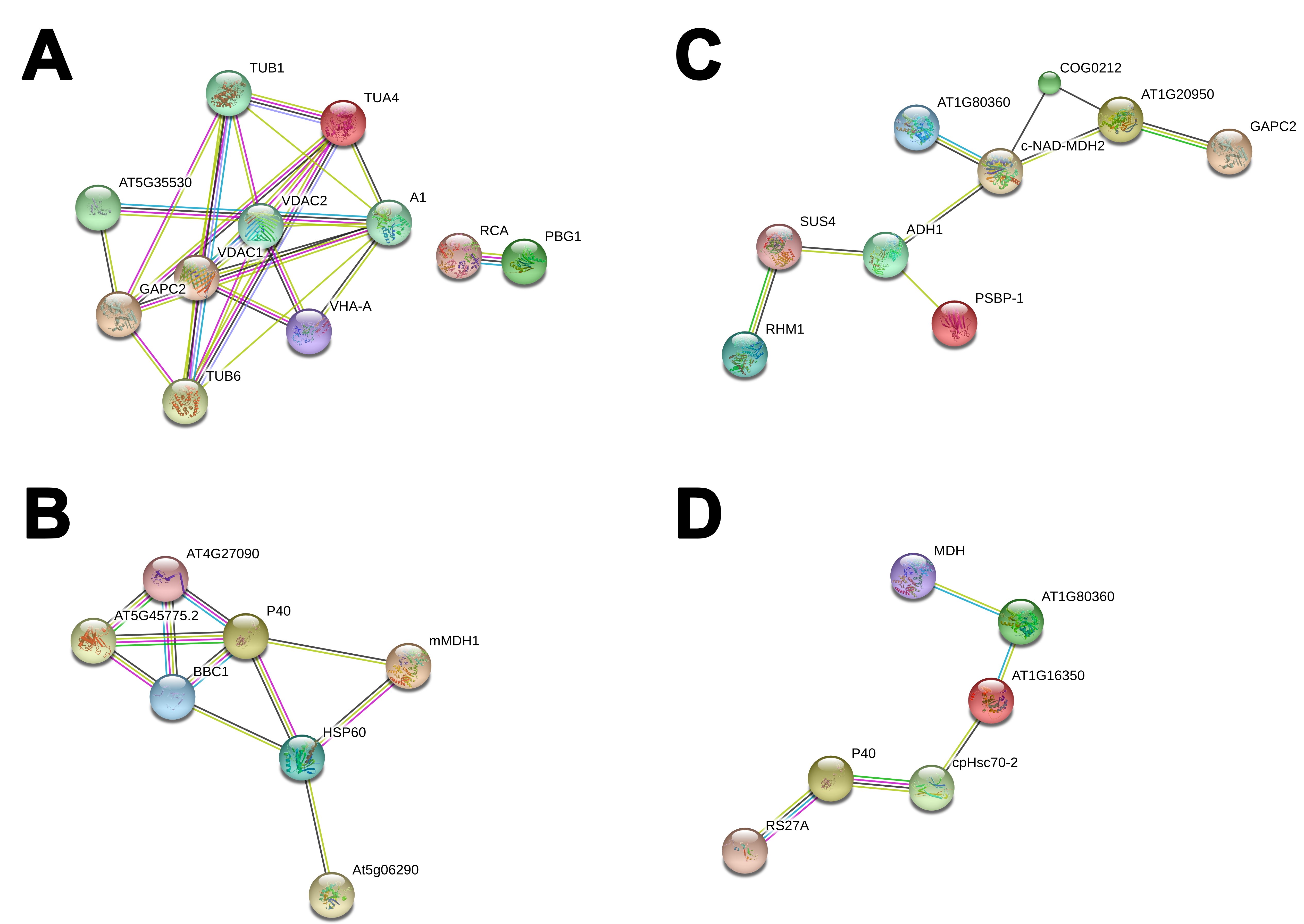

Supplement: Supplementary file 7 — Additional file 7. Differentially expressed proteins of TSH1188 and Catongo during interaction with M. perniciosa subjected PPI analysis. Networks of up regulated (A) and down regulated (B) proteins in TSH1188 at 72HAI. Networks of up regulated (C) and down regulated (D) proteins in Catongo at 72HAI. Network nodes represent proteins in which each node represents all the protein by a single protein-coding gene locus. Small nodes indicate proteins of unknown 3D structure, large nodes indicate proteins which 3D structures are known or predict (can be visualized by close-up the nodes). Different line colors indicate the types of evidence for the associations. Query proteins not connected with network were removed for better visualization. [file 12870_2019_2170_MOESM7_ESM.png]
