# Supplementary material for: The pathogen Moniliophthora perniciosa promotes differential proteomic modulation of cacao genotypes with contrasting resistance to witches´ broom disease
Source: BMC Plant Biol. 2020 Jan 2;20:1. doi: 10.1186/s12870-019-2170-7 (PMC6941324; doi:10.1186/s12870-019-2170-7)
Supplement: Supplementary file 9 — Additional file 9. Detailed protein extraction method. [file 12870_2019_2170_MOESM9_ESM.docx]

**Protein extraction**

The meristems (shoot apex) were submitted to protein extraction using chemical and physical methods to optimize the protein yield, following the protocol developed by Pirovani and colleagues (2008) [1] with modifications. The meristems were macerated in presence of liquid nitrogen and Polyvinylpolypyrrolidone (PVPP). The plant material (mean 0.214g for each treatment) were washed four times with cold acetone 100% + β-mercaptoetanol 0.07%, and centrifuged (14,000 rpm, 10 min at 4ºC). The precipitated was dehydrated in laminar flow chamber at ambient temperature for 30 min, and washed 3 times in acetone + Trichloroacetic acid (TCA) 10% + β-mercaptoetanol 0.07 %, followed by homogenization in vortex and sonication (4 cycles of 10 seconds alternated with 10 seconds of rest, with amplitude of 70%). The *pellet* was washed 3 times with water + TCA 10% + β-mercaptoetanol 0.07 % solution, homogenized, sonicated and centrifuged at the same conditions previously cited, and washed twice with acetone 80% + β-mercaptoetanol 0.07 %. The obtained O *pellet* was dehydrated in laminar flow chamber at ambient temperature.

SDS/fenol extraction step: A volume of 3 mL of SDS buffer (Tris-HCl 100 mmol.L^-1^, pH 8, sacarose 30 %, SDS 2 % and β-mercaptoetanol 5 %) was added to the pellet and rapidly homogenized using sonication (4 cycles of 10 seconds alternated with 10 seconds of rest, with amplitude of 50 %), 3 mL of phenol 99% buffered in pH 8 with Tris were added. Homogenization was carried out in vortex for 1 min and allowed to rest for 30 min. After, a centrifugation step (10,000 rpm, 10 min at 4ºC) was carried out. The phenolic phase was removed and transferred to a new tube. To the phenolic phase were added 5 volumes of ammonium acetate 0.1 mmol.L^-1^ in cold methanol and stored at -20 ºC *overnight*. The proteins were recovered by centrifugation, and then washed twice with ammonium acetate 0.1 mmol.L^-1^ in cold methanol and the pellet was homogenized by vortex and centrifuged at each wash under the same conditions cited above. After that, the pellet was washed twice with acetone 80% at same conditions cited above. The pellet was then dehydrated at ambient temperature and rehydrated in 600µl of rehydration buffer (Ureia 7M, Tioureia 2 M, CHAPS 2 %, Bromophenol blue 0.002%). The proteins were solubilized in buffer overnight inside the refrigerator and then stored at -20 °C until use.

[1] Pirovani CP, Carvalho HA, Machado RC, Gomes DS, Alvim FC, Pomella AW, Gramacho KP, Cascardo JC, Pereira GA, Micheli F. Protein extraction for proteome analysis from cacao leaves and meristems, organs infected by Moniliophthora perniciosa, the causal agent of the witches’ broom disease. Electrophoresis. 2008;29:391–401. doi: 10.1002/elps.200700743.
